# Fully Affine Invariant Methods for Cross-Session Registration of Calcium Imaging Data

**DOI:** 10.1523/ENEURO.0054-20.2020

**Published:** 2020-08-07

**Authors:** Chunyue Li, Xiaofeng Yang, Ya Ke, Wing-Ho Yung

**Affiliations:** School of Biomedical Sciences and Gerald Choa Neuroscience Centre, Faculty of Medicine, The Chinese University of Hong Kong, Shatin, Hong Kong 999077

**Keywords:** cross-session field-of-view alignment, fully affine invariant approach, two-photon calcium microscopy

## Abstract

*In vivo* two-photon microscopy permits simultaneous recording of the activity of the same neuronal population across multiple sessions in days or weeks, which is crucial for addressing many fundamental questions of neuroscience. The field-of-view (FOV) alignment is a necessary step for identifying the same neurons across multiple imaging sessions. Accurate FOV alignment becomes challenging in the situations of image blurring, insufficient common neurons, or uneven background brightness. The existing methods largely fail to align FOV pairs in these situations. The fully affine invariant approach has been applied in computer vision to register real scene images with different backgrounds. However, its performance in calcium imaging data is unknown. We explored the feasibility of using the fully affine invariant approach to align calcium FOV images across multiple sessions by examining the performance of five methods. Further, we compared their performance with common feature-based methods as well as some classical methods with or without adaptive contrast enhancement. Using cellular resolution calcium imaging data recorded from two areas of the mouse motor cortex over weeks, we show that all fully affine invariant methods provide more accurate FOV alignment results than other methods in general and in the case of a few common neurons identified, uneven background brightness or image blurring. This study demonstrated the feasibility and reliability of the fully affine invariant methods in cross-session FOV alignment. These methods could be useful for neuroscience research, especially on questions that involve experience-dependent plasticity spanning over days or weeks.

## Significance Statement

Field-of-view (FOV) alignment is challenging when neurons collected in two sessions are not one-to-one mapped or calcium data are recorded under different imaging parameters and brain states. For the first time, we explored the feasibility of using the fully affine invariant methods to align calcium FOV images across multiple sessions and compared their performance with many conventional methods and their variations. We demonstrate that fully affine invariant methods outperform other conventional methods and are robust under unfavorable conditions. Our work is important for studies on experience-dependent processes, such as learning and memory. Moreover, although fully affine invariant methods are conducted on two-photon calcium imaging data, these methods should be promising in FOV alignment of one-photon or widefield fluorescence microscopy.

## Introduction

*In vivo* two-photon calcium imaging on rodents is a crucial technique for studying many fundamental questions in the field of neuroscience such as visual processing and motor control (Han et al., 2019; Hwang et al., 2019; Stringer et al., 2019). This technique is particularly useful for studying brain mechanisms of learning and memory as it allows researchers to record experience-dependent changes of neurons over extended periods of time in awake behaving animals (Grewe et al., 2017; Pakan et al., 2018; Namboodiri et al., 2019; Wagner et al., 2019).

To chronically follow neurodynamics, the same group of neurons must be reliably registered across multiple sessions (or days). The field-of-view (FOV) alignment is a necessary step for cell registration (Kaifosh et al., 2014; Sheintuch et al., 2017; Giovannucci et al., 2019). However, several factors could induce uncertainty and potential errors in the FOV alignment, rendering it a rather challenging step for cell registration. First, manual head-fixing in each recording session can easily lead to viewing angle change in addition to X-Y plane translation and rotation, making a one-to-one mapping of neural identity not necessarily attainable. Second, for long-term recording, the quality of the microscopic image may decrease because of photobleaching, phototoxicity, and brain state change (Lichtman and Conchello, 2005), necessitating the use of different acquisition parameters, which could result in different background intensities and signal-to-noise ratios across sessions. Lastly, for rodents, the common problem of dural regrowth increases with the number of sessions, which reduces optical transparency and leads to image blurring (Heo et al., 2016).

In the past, many efficient methods have been used to register the calcium FOV across multiple sessions. These include classical intensity-based methods, such as TurboReg (Thévenaz et al., 1998), Lucas-Kanade (LK; Baker and Matthews, 2004), and enhanced correlation coefficient (ECC; Evangelidis and Psarakis, 2008). There are also approaches like MOCO (Dubbs et al., 2016) and non-rigid NoRMCorre (Pnevmatikakis and Giovannucci, 2017) used by the popular CaImAn toolbox (Giovannucci et al., 2019). Recently, feature-based approach, such as scale-invariant feature transform (SIFT; Lowe, 2004), Speeded Up Robust Features (SURF; Bay et al., 2008), Accelerated-KAZE (AKAZE; Alcantarilla and Solutions, 2011), Binary Robust Invariant Scalable Keypoints (BRISK; Leutenegger et al., 2011), and Oriented FAST and Rotated BRIEF (ORB; Rublee et al., 2011) have also been used in microscopic image alignment (Stanciu et al., 2010; Ünay and Stanciu, 2018; Chen et al., 2019). However, because of different reasons, these techniques could fail in the situations of image blurring, insufficient common neurons or uneven background brightness. Given the limitations, a new approach in FOV alignment that could achieve more robust results is much warranted.

ASIFT (Yu and Morel, 2009, 2011) is a fully affine invariant method. It simulates all possible affine distortions caused by the viewing angle changes and then applying the SIFT method to compare keypoints detected from all the simulated images. ASIFT can acquire more keypoints than SIFT even in the situation of negligible or moderate camera view angle change (Yu and Morel, 2011), which means that ASIFT could be applicable for calcium FOV alignment. Moreover, the principles of ASIFT, i.e., matching keypoints detected in both original images as well as affine simulations, can be extended to other similar invariant matching methods, such as SURF, AKAZE, ORB, and BRISK, making them potential solutions for calcium activity FOV alignment. However, the performance of ASIFT and extended fully affine feature-based methods [Affine-SURF (ASURF), Affine-AKAZE (AAKAZE), Affine-BRISK (ABRISK), and Affine-ORB (AORB)] on calcium imaging data is unknown.

In this study, we investigated the performance of ASIFT, ASURF, AAKAZE, ABRISK, and AORB on cross-session FOV alignment of calcium imaging data. By using L1-norm, we decreased their unreliability caused by the random sample consensus (RANSAC; Fischler and Bolles, 1981). Further, we compared their performance with general feature-based methods, i.e., SIFT, SURF, AKAZE, BRISK, and ORB, widely used methods, i.e., LK, ECC, MOCO, TurboReg, and NoRMCorre, as well as these widely used methods combined with a contrast-limited adaptive histogram equalization (CLAHE; Reza, 2004). For convenience, the four groups of methods are named as the fully affine invariant group, feature-based group, the conventional group, and CLAHE-based conventional group, respectively. We found that the fully affine invariant group is superior to other methods even in the situation of image blurring, insufficient common neurons, and uneven background brightness. As far as we know, this is the first study that demonstrated the feasibility of the fully affine invariant approach in cross-session FOV alignment of calcium imaging data.

## Materials and Methods

### Data collection

**Figure 1.**
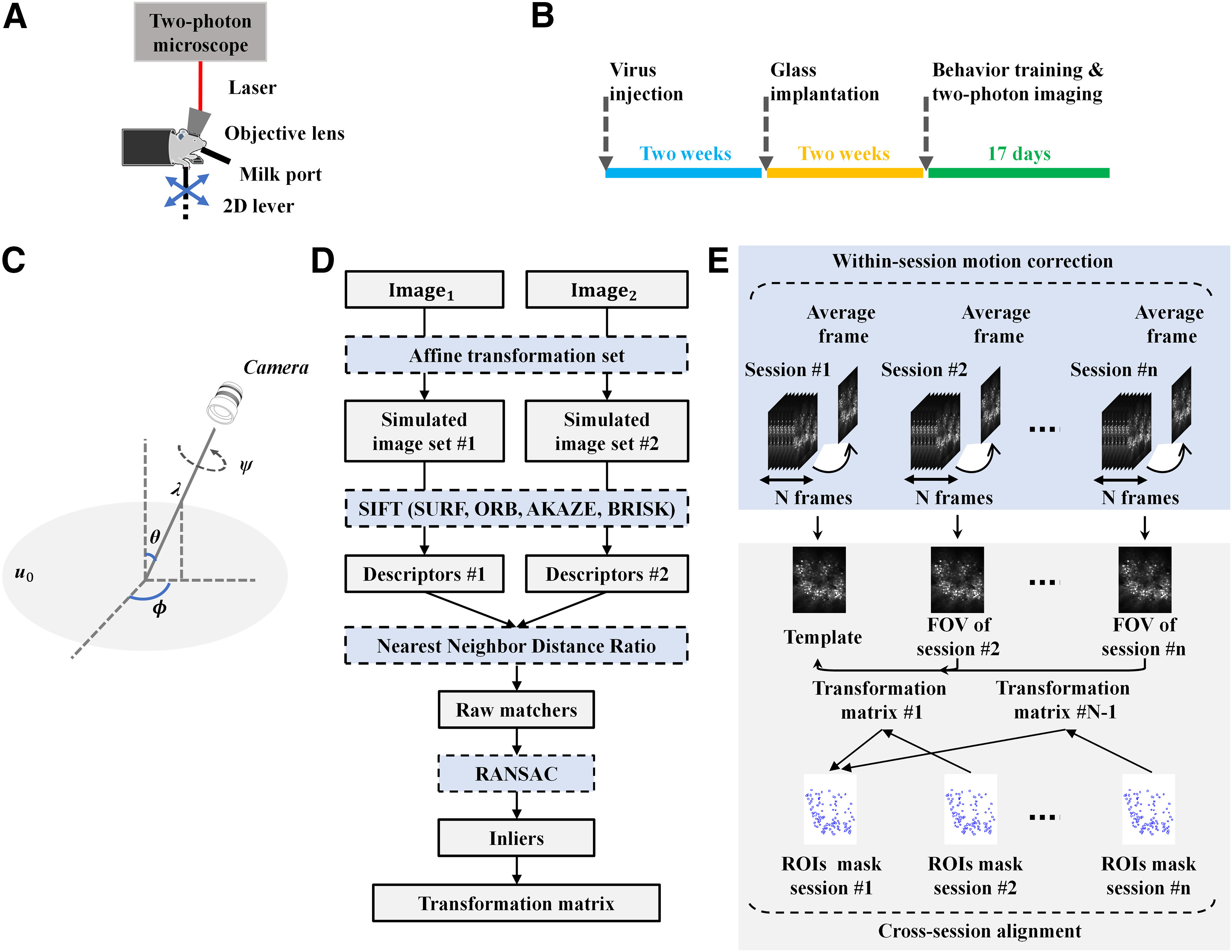
Illustration of experimental design and the proposed FOV alignment approach. ***A***, *In vivo* set up for two-photon imaging data collection. ***B***, Experimental procedure. The GCaMP6f was injected into the RFA and CFA of the layer 2/3 motor cortex. Two weeks later, a cranial window surgery was conducted above the RFA and CFA. Behavioral training and two-photon imaging recording began two weeks after the window surgery. The mouse received one session per day and 17 sessions in total. ***C***, Geometric interpretation of the affine decomposition. *λ* and *ψ* are the zoom factor and the rotation angle of the camera around the optical axis respectively. *ϕ* and *θ* corresponds to the longitude and latitude angles of the optical axis. ***u*_0_**represents the frontal view of the flat object. ***D***, Generic phases of the ASIFT method. **Image_1_** and **Image_2_** were individually transformed by simulating a large set of affine distortions caused by the change of longitude *ϕ* and latitude *θ*. Then, SIFT was used to detect and describe the keypoints on every simulated image. NNDR was used to match the keypoints. RANSAC was used to exclude outliers from initial matches. The remaining inliers were used to estimate the transformation matrix. SIFT was replaced by SURF, AKAZE, BRISK, and ORB to achieve ASURF, AAKAZE, ABRISK, and AORB. ***E***, Outline of the FOV alignment procedure. TurboReg was used to process within-session motion artifacts. The motion-corrected imaging session was averaged and normalized to get the corresponding FOV image. The FOV image of the first session was used as the template, and FOV images of all other sessions were aligned to it. The alignment was achieved by fully affine invariant methods (ASIFT, ASURF, AAKAZE, ABRISK, AORB), the feature-based methods (SIFT, SURF, AKAZE, BRISK, ORB), the conventional methods (LK, ECC, MOCO, TurboReg, NoRMCorre), and the CLAHE-based conventional methods (LK-CLAHE, ECC-CLAHE, MOCO-CLAHE, TurboReg-CLAHE, NoRMCorre-CLAHE).

Calcium imaging data were collected from layer 2/3 in the rostral forelimb area (RFA) and caudal forelimb area (CFA) of the primary motor cortex with a custom-built *in vivo* two-photon microscope while a male C57 mouse learned a two-dimensional (2D) lever reaching task (Fig. 1*A*). GCaMP6f was injected into the RFA and the CFA to express GCaMP6f in all neuron types. Two weeks after virus injection, the skull located above the recording areas was removed, and the brain surface was covered with a glass coverslip. Behavioral training and two-photon imaging began two weeks after the window surgery. The mouse received one session (∼100 trials) per day and 17 sessions in total. Each FOV had a size of 512 × 512 pixels and the acquisition frequency was 15 Hz. The experimental procedure is summarized in Figure 1*B*. All animal procedures were performed in accordance with the Chinese University of Hong Kong animal care committee’s regulations.

### Regions of interest (ROIs) mask

Neuron detection and FOV alignment are two necessary steps for cell registration. Neuron detection aims to obtain a ROIs mask for each imaging session. The ROIs mask includes coordinates or spatial footprints of all active neurons that appeared in one session. FOV alignment seeks to transform the ROIs mask from different sessions into one single coordinate system. Cellpose (Stringer et al., 2020) was applied to the mean calcium image of each session to get the corresponding ROIs mask. Cellpose can precisely segment neurons of various types and without the needs of model retraining or parameter adjustments. Since dendritic spines can easily be misdetected as neurons, we excluded them by requiring that the ROI mask of individual neurons should contain at least 60 pixels. Then, each ROIs mask was binaried so that the pixel value within a neuron is 255 and the pixel of other places is set as 0. In this study, the paired raw ROIs masks were represented as $\left\{ROIs_{Mask}{\;}_{n}^{raw},ROIs_{Mask}{\;}_{template}^{raw}\right\}$. Here, the template was defined as the first session; *n* defined the session index. In addition, the neurons that existed in both template session and each registered session were manually selected and were saved in $\left\{ROIs_{Mask}{\;}_{n}^{common},ROIs_{Mask}{\;}_{template}^{common}\right\}$, which was used to evaluate the performance of different alignment methods.

### ASIFT method

Generally, a digital image ***u*** of a flat physical object ***u*_0_** can be expressed as ***u*** = ***G*_1_*Au*_0_**. Here, ***G*_1_** is a Gaussian kernel modeling the optical blur, and ***A*** is a planar protective map. Moreover, if the shape of ***u*_0_** is locally smooth, the protective map ***A*** can be simplified to an affine map. Therefore, the local deformation model is ***u***(*x, y*) → ***u***(*ax* + *by* + *e, cx* + *dy* + *f*) in each image region (*x*, *y*), where $A=\left[\begin{array}{ll} a & b\\ c & d \end{array}\right]$ represents an affine map and $\left(\begin{array}{l} e\\ f \end{array}\right)$ represents the translation. Further, the affine map ***A*** with positive determinant can be decomposed as

$$A=\lambda \left[\begin{array}{ll} \text{cos}\psi & -\text{sin}\psi \\ \text{sin}\psi & \text{cos}\psi \end{array}\right]\left[\begin{array}{ll} t & 0\\ 0 & 1 \end{array}\right]\left[\begin{array}{ll} \text{cos}\phi & -\text{sin}\phi \\ \text{sin}\phi & \text{cos}\phi \end{array}\right],$$

where *λ* and *ψ* denote the zoom factor and the rotation angle of camera around optical axis, respectively; *ϕ* and *θ* = arccos(1/*t*) represents the longitude and latitude angles of the optical axis, respectively. Figure 1*C* shows the geometric interpretation of the affine decomposition.

Suppose **Image_1_**= ***G*_1_*A*_1_*u*_0_** and **Image_2_**= ***G*_1_*A*_2_*u*_0_** are two digital images of the same object ***u*_0_** taken with different affine map ***A*_1_** and ***A*_2_**, respectively. To register **Image_1_** and **Image_2_**, each of them was individually transformed by simulating a large set of affine distortions caused by the change of longitude *ϕ* and latitude *θ*. The simulation was achieved by varying *ϕ* with the change of *θ*, with the step Δ*ϕ* = (72°/*t*), *t* = (1/cos*θ*), *ψ* ∈ [0, π], and *θ* ∈ [–π/2, π/2]. This operation enables ASIFT to be invariant to viewing angle changes. Then, SIFT was used to detect and describe the keypoints on simulated images. The keypoints detected by SIFT are invariant to translation, rotation, and scaling. Therefore, ASIFT becomes a fully invariant method by combining SIFT and the affine simulation.

In this study, SIFT was replaced by SURF, AKAZE, BRISK, and ORB to achieve ASURF, AAKAZE, ABRISK, and AORB. After feature detection and description, the nearest neighbor distance ratio (NNDR) was used to match the keypoints detected in two simulated image sets (Lowe, 2004). The threshold ratio of NNDR was set as 0.75. Then, outliers were further excluded from initial matches by using RANSAC with 150,000 iterations and 99.9% confidence. The transformation matrix was estimated using the remaining inliers. Figure 1*D* presented the procedure of the fully affine invariant approach.

### FOV alignment

FOV alignment included two steps: within-session motion correction and cross-session alignment. The intensity-based method TurboReg was used to process within-session motion artifacts because calcium imaging data collected within each session have a similar intensity distribution. Specifically, for each session, the average image was taken as the template, and all other calcium frames within this session were registered to the template. Then, the motion-corrected calcium session was averaged and normalized to get the corresponding FOV image. For cross-session alignment, the FOV image of the first session was used as the template, and FOV images of all other sessions were aligned to it. The alignment was achieved by fully affine invariant methods (ASIFT, ASURF, AAKAZE, ABRISK, AORB), the feature-based methods (SIFT, SURF, AKAZE, BRISK, ORB), the conventional methods (LK, ECC, MOCO, TurboReg, NoRMCorre), and the CLAHE-based conventional methods (LK-CLAHE, ECC-CLAHE, MOCO-CLAHE, TurboReg-CLAHE, NoRMCorre-CLAHE). The derived transformation matrix was applied on $ROIs_{Mask}{\;}_{n}^{common}$, where *n* defines the session index. The correlation between $ROIs_{Mask}{\;}_{n}^{common}$ and $ROIs_{Mask}{\;}_{template}^{common}$ was used to evaluate the performance of these methods. Specifically, the 2D ROIs masks were first reshaped into 1D vectors, then the Pearson’s linear correlation coefficient between these vectors was calculated. The ROI masks are binary images containing only 0 or 255. Therefore, the higher the correlation coefficient, the more similar the ROIs masks. The FOV alignment procedure was summarized in Figure 1*E*.

### Reliability improvement of the fully affine invariant group and the feature-based group

NNDR was used to find initial matches of keypoints for both the fully affine invariant group and the feature-based group. Further, RANSAC was used to exclude outliers from the initial matches. However, in theory, RANSAC cannot ensure to eliminate all outliers and preserve all inliers (Chen et al., 2019). If some important inliers are missed, the registered image will be distorted. Moreover, RANSAC could produce different results each time because of its randomness (Hast et al., 2013). To obtain reliable and reproducible results for both two groups, we repetitively run the NNDR and RANSAC 100 times for each image pair, then choose the transformation matrix which minimizes the L1-norm of the intensity difference of where neuron appears between $\left\{ROIs_{Mask}{\;}_{n}^{raw},ROIs_{Mask}{\;}_{template}^{raw}\right\}$.

### CLAHE for the conventional group

Uneven background brightness of the FOV image will decrease the performance of the methods in the conventional group. Therefore, CLAHE was used to enhance the contrast of FOV images. Specifically, CLAHE divided an image into multiple non-overlapping blocks. For each block, the histogram was clipped and redistributed to avoid overenhancement. Further, bilinear interpolation was used for adjacent blocks to avoid the appearance of block artifacts. After contrast enhancement, methods in the conventional group were applied on CLAHE adjusted FOV images, and their results were compared with the fully affine invariant group.

### Image quality metrics

A sharpness metrics was used to evaluate image blurriness. Image sharpness is defined as the ratio of high-frequency components above a certain threshold to all pixels in an image (De and Masilamani, 2013). The lower the value, the more blurred the FOV image. The high-frequency threshold was calculated by *M*/1000. *M* is the maximum value of the centered Fourier spectrum of the FOV template. It has been shown that this particular threshold value gives a fairly accurate sense of image quality (De and Masilamani, 2013).

The other metrics is the number of neurons common to each $ROIs_{Mask}{\;}_{n}^{raw}$ and $ROIs_{Mask}{\;}_{template}^{raw}$. This metrics measures the content similarity of the FOV image pairs. Usually, the higher the similarity, the more the common neurons.

### Codes and computational hardware

Opencv-contrib-python3.4.2.17 (Bradski, 2000) was used to perform the function of CLAHE, feature-based methods, and fully affine invariant methods. Python package pyStackReg (https://pypi.org/project/pystackreg/) was used for TurboReg. MATLAB 2020a was used to run LK, ECC, and non-rigid NoRMCorre. Codes of LK and ECC were from the online IAT toolbox (Evangelidis, 2013). Codes of non-rigid NoRMCorre were public online. Fiji was used for MOCO.

All above codes were performed on a Windows 10-based laptop equipped with an Intel i7-5500U CPU running at 2.40 GHz and 16GB RAM.

### Code accessibility

The code described in the paper is freely available online at https://github.com/chunyueli/FAIMCalcium. The code is available as Extended Data.

10.1523/ENEURO.0054-20.2020.ed Extended Data. A zip file (named “data_code.zip”), including PyPI package (“FAIM_package” folder), example FOV images (within “examples” folder), and codes used to reproduce all results (within “AffineCa2p_reproduce_results” folder) were submitted as Extended Data. Each folder contains a readme file. Download Extended Data, EPS file.

## Results

### *In vivo* calcium imaging data

**Figure 2.**
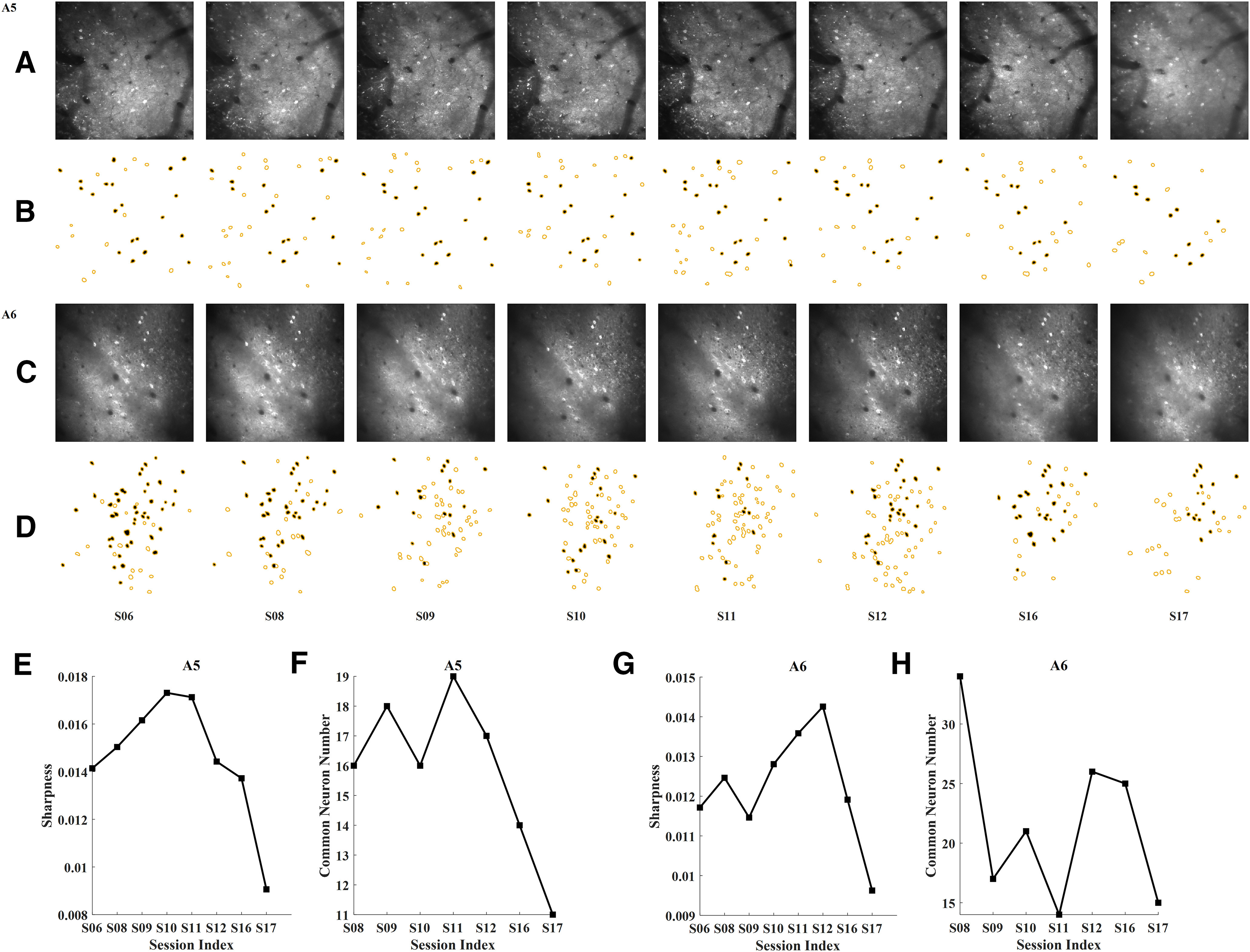
Basic information of the collected FOV images. ***A***, The FOV image of each session from RFA, labeled as A5. ***B***, The ROIs mask of each session from A5. ***C***, The FOV image of each session from CFA, labeled as A6. ***D***, The ROIs mask of each session from A6. The yellow circles represent the detected neurons in each FOV image. The yellow circles filled with black color represent the neurons common to each $ROIs_{Mask}{\;}_{n}^{raw}$ and $ROIs_{Mask}{\;}_{template}^{raw}$. *n* defines the session index. S is short for session. ***E***, The sharpness metrics of each session for A5. The lower the value, the more blurred the FOV image. ***F***, Number of neurons common to each $ROIs_{Mask}{\;}_{n}^{raw}$ and $ROIs_{Mask}{\;}_{template}^{raw}$ for A5. ***G***, The sharpness metrics of each session for A6. ***H***, Number of neurons common to each $ROIs_{Mask}{\;}_{n}^{raw}$ and $ROIs_{Mask}{\;}_{template}^{raw}$ for A6.

Calcium imaging data of eight sessions with irregular session-interval collected from two cortical areas RFA and CFA (labeled as A5 and A6, respectively) of a mouse were used in this study. For each imaging area, the FOV image of the first recorded session (labeled as S06) was selected as the template. Figure 2*A*,*C* shows the FOV image of each session from A5 and A6. As can be seen, the background brightness of the FOV images were uneven and varied across sessions. The yellow circles in Figure 2*B*,*D* represent the detected neurons in $ROIs_{Mask}{\;}_{n}^{raw},n\in \left(S06,S08,S09,S10,S11,S12,S16,S17\right)$. The yellow circles filled with black color represent the neurons common to each $ROIs_{Mask}{\;}_{n}^{raw}$ and $ROIs_{Mask}{\;}_{template}^{raw}$. As can be seen, the common neurons varied across sessions, so there was no one-to-one mapping between the template session and the registered session.

### Image quality evaluation

Figure 2*E*,*G* shows the sharpness metrics of each FOV image from A5 and A6. The results indicate that session 17 (S17) has the minimum sharpness value for both areas. Thus, S17 was more blurred than other sessions for both areas. Figure 2*F*,*H* displays the number of neurons common to each $ROIs_{Mask}{\;}_{n}^{raw}$ and $ROIs_{Mask}{\;}_{template}^{raw}$ for the two areas. The results showed that S16 and S17 had smaller number of common neurons than other sessions of A5. S11 and S17 had smaller number of common neurons than other sessions of A6.

### Comparison between the fully affine invariant group and the feature-based group

Tables 1, 2 show the quantitative comparison between the fully affine invariant group and the feature-based group of the A5 and A6, respectively. As can be seen, fully affine invariant methods generated more inliers than feature-based methods on sessions that had a low sharpness metrics or small common neuron number. For instance, the inliers of ASIFT, ASURF, AAKAZE, ABRISK, and AORB were 20, 20, 13, 12, and 13 for S17 of A5 while the inliers obtained by SIFT, SURF, AKAZE, BRISK, and ORB were 4, 6, 5, 4, and 7 for S17 of A5.

**Table 1.** Quantitative comparison between the fully affine invariant group and the feature-based group with respect to area A5

Methods	Features detectedin image pair		Inliers	Matchedfeatures	Inlierratio	Methods	Features detectedin image pair		Inliers	Matchedfeatures	Inlierratio
	Image	Template(S06)					Image	Template(S06)			
Image pair #1 of A5 (S08, S06)											
ASIFT	10636	9524	95	241	0.39	SIFT	629	565	10	17	0.59
ASURF	20012	18698	131	427	0.30	SURF	1258	1174	17	42	0.40
AAKAZE	3120	2893	55	89	0.62	AKAZE	236	209	11	18	0.61
ABRISK	8677	7667	32	57	0.56	BRISK	918	894	10	17	0.59
AORB	13927	13427	31	100	0.31	ORB	500	500	9	11	0.82
Image pair #2 of A5 (S09, S06)											
ASIFT	11357	9524	82	195	0.42	SIFT	690	565	12	22	0.55
ASURF	20398	18698	98	397	0.25	SURF	1346	1174	17	36	0.47
AAKAZE	3267	2893	47	91	0.52	AKAZE	269	209	10	16	0.63
ABRISK	9830	7667	50	80	0.63	BRISK	1169	894	15	26	0.58
AORB	14111	13427	43	96	0.45	ORB	500	500	9	17	0.53
Image pair #3 of A5 (S10, S06)											
ASIFT	12935	9524	41	174	0.23	SIFT	771	565	10	23	0.43
ASURF	22158	18698	49	408	0.12	SURF	1466	1174	7	45	0.16
AAKAZE	3697	2893	29	67	0.43	AKAZE	270	209	8	15	0.53
ABRISK	10848	7667	21	41	0.51	BRISK	1260	894	10	15	0.67
AORB	14387	13427	18	103	0.17	ORB	500	500	4	8	0.50
Image pair #4 of A5 (S11, S06)											
ASIFT	13833	9524	61	163	0.37	SIFT	850	565	9	26	0.35
ASURF	22345	18698	83	407	0.20	SURF	1446	1174	8	32	0.25
AAKAZE	3753	2893	30	76	0.39	AKAZE	291	209	7	10	0.70
ABRISK	11149	7667	18	44	0.41	BRISK	1243	894	11	18	0.61
AORB	14514	13427	20	105	0.19	ORB	500	500	4	5	0.80
Image pair #5 of A5 (S12, S06)											
ASIFT	10106	9524	71	176	0.40	SIFT	588	565	13	20	0.65
ASURF	20415	18698	71	404	0.18	SURF	1293	1174	7	35	0.20
AAKAZE	3239	2893	47	90	0.52	AKAZE	240	209	15	19	0.79
ABRISK	8477	7667	29	50	0.58	BRISK	874	894	6	9	0.67
AORB	14062	13427	56	148	0.38	ORB	500	500	13	25	0.52
Image pair #6 of A5 (S16, S06)											
ASIFT	9751	9524	17	114	0.15	SIFT	613	565	4	9	0.44
ASURF	20095	18698	28	296	0.095	SURF	1275	1174	6	24	0.25
AAKAZE	3269	2893	12	39	0.31	AKAZE	240	209	5	15	0.33
ABRISK	7416	7667	13	40	0.33	BRISK	811	894	4	8	0.50
AORB	13690	13427	16	97	0.16	ORB	500	500	8	24	0.33
Image pair #7 of A5 (S17, S06)											
ASIFT	3945	9524	20	69	0.29	SIFT	202	565	4	5	0.80
ASURF	12914	18698	20	242	0.08	SURF	649	1174	6	27	0.22
AAKAZE	1802	2893	13	33	0.39	AKAZE	128	209	5	8	0.63
ABRISK	3092	7667	12	26	0.46	BRISK	201	894	4	4	1.00
AORB	9454	13427	13	85	0.15	ORB	498	500	7	14	0.50
Mean values for all image pairs
ASIFT	10366.14	9524	55.29	161.71	0.34	SIFT	620.43	565	8.86	17.43	0.51
ASURF	19762.43	18698	68.57	368.71	0.19	SURF	1247.57	1174	9.71	34.43	0.28
AAKAZE	3163.86	2893	33.28	69.29	0.48	AKAZE	239.14	209	8.71	14.43	0.60
ABRISK	8498.43	7667	25	48.26	0.52	BRISK	925.14	894	8.57	13.86	0.62
AORB	13449.29	13427	28.14	104.86	0.27	ORB	499.71	500	7.71	14.85	0.52

**Table 2.** Quantitative comparison between the fully affine invariant group and the feature-based group with respect to area A6

Methods	Features detectedin image pair		Inliers	Matchedfeatures	Inlierratio	Methods	Features detectedin image pair		Inliers	Matchedfeatures	Inlierratio
	Image	Template(S06)					Image	Template(S06)			
Image pair #1 of A6 (S08, S06)											
ASIFT	6485	6184	1236	1412	0.88	SIFT	431	444	117	143	0.82
ASURF	13192	12398	1705	2351	0.73	SURF	832	843	123	162	0.76
AAKAZE	2864	2586	840	1179	0.71	AKAZE	208	191	95	108	0.88
ABRISK	5221	5164	777	880	0.88	BRISK	417	432	86	93	0.92
AORB	13177	12917	1467	1858	0.79	ORB	500	500	101	127	0.80
Image pair #2 of A6 (S09, S06)											
ASIFT	6611	6184	191	365	0.52	SIFT	477	444	30	44	0.68
ASURF	13411	12398	322	734	0.44	SURF	886	843	30	59	0.51
AAKAZE	2530	2586	351	490	0.72	AKAZE	219	191	37	56	0.66
ABRISK	4636	5164	203	296	0.69	BRISK	419	432	30	43	0.70
AORB	12477	12917	335	555	0.60	ORB	500	500	32	52	0.62
Image pair #3 of A6 (S10, S06)											
ASIFT	7681	6184	469	602	0.78	SIFT	521	444	53	66	0.80
ASURF	12154	12398	554	880	0.63	SURF	844	843	62	86	0.72
AAKAZE	2837	2586	422	546	0.77	AKAZE	263	191	48	61	0.79
ABRISK	6476	5164	459	548	0.84	BRISK	643	432	70	74	0.95
AORB	13486	12917	784	1027	0.76	ORB	500	500	61	75	0.81
Image pair #4 of A6 (S11, S06)											
ASIFT	8556	6184	132	273	0.48	SIFT	581	444	30	40	0.75
ASURF	13982	12398	195	502	0.39	SURF	931	843	35	53	0.66
AAKAZE	3033	2586	200	299	0.67	AKAZE	271	191	31	47	0.66
ABRISK	7262	5164	159	239	0.67	BRISK	720	432	32	42	0.76
AORB	14158	12917	255	396	0.64	ORB	500	500	26	41	0.63
Image pair #5 of A6 (S12, S06)											
ASIFT	9027	6184	426	624	0.68	SIFT	651	444	58	79	0.73
ASURF	13988	12398	448	772	0.58	SURF	935	843	57	85	0.67
AAKAZE	3031	2586	278	403	0.69	AKAZE	251	191	52	66	0.79
ABRISK	7478	5164	365	452	0.81	BRISK	662	432	50	59	0.85
AORB	13889	12917	553	750	0.74	ORB	500	500	62	77	0.81
Image pair #6 of A6 (S16 S06)											
ASIFT	6248	6184	392	532	0.74	SIFT	388	444	49	56	0.88
ASURF	12517	12398	399	681	0.59	SURF	782	843	53	75	0.71
AAKAZE	2424	2586	338	359	0.94	AKAZE	197	191	47	56	0.84
ABRISK	4596	5164	240	271	0.89	BRISK	371	432	41	53	0.77
AORB	12004	12917	447	556	0.80	ORB	500	500	49	57	0.86
Image pair #7 of A6 (S17, S06)											
ASIFT	4343	6184	80	140	0.57	SIFT	279	444	17	22	0.77
ASURF	11170	12398	82	291	0.28	SURF	704	843	13	30	0.43
AAKAZE	1885	2586	62	92	0.67	AKAZE	140	191	16	19	0.84
ABRISK	3449	5164	43	57	0.75	BRISK	254	432	19	20	0.95
AORB	10545	12917	76	143	0.53	ORB	500	500	21	34	0.62
Mean values for all image pairs											
ASIFT	6993	6184	418	564	0.74	SIFT	475.43	444	50.57	64.29	0.79
ASURF	12916.29	12398	529.29	887.29	0.60	SURF	844.86	843	53.29	78.57	0.68
AAKAZE	2657.71	2586	355.86	481.14	0.74	AKAZE	221.29	191	46.57	59	0.79
ABRISK	5588.29	5164	320.86	391.86	0.82	BRISK	498	432	46.86	54.86	0.85
AORB	12819.42	12917	559.57	755	0.74	ORB	500	500	50.29	66.14	0.76

**Figure 3.**
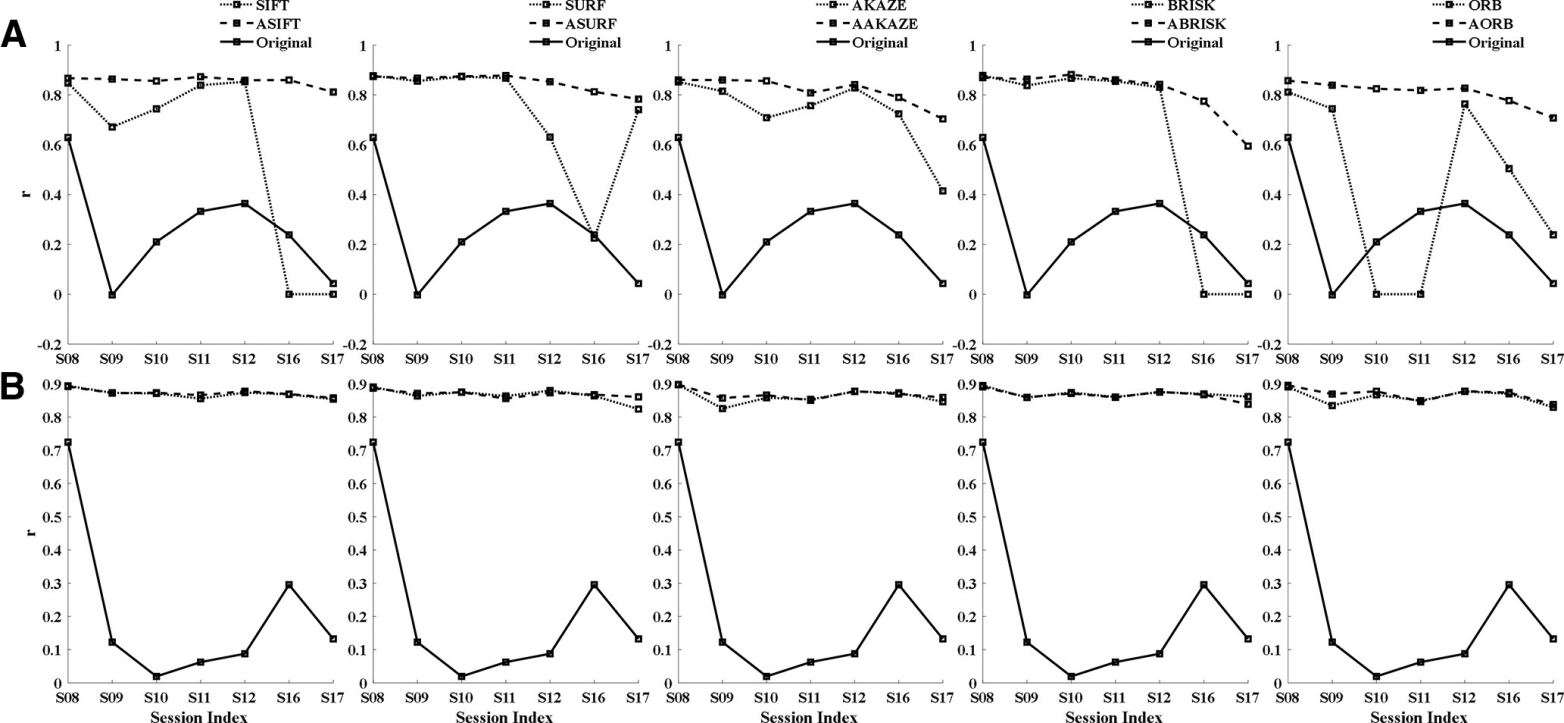
Comparison of performance between the fully affine invariant group and the feature-based group. The correlation between $ROIs_{Mask}{\;}_{template}^{common}$ and the registered $ROIs_{Mask}{\;}_{n}^{common},n\in \left(S08,S09,S10,S11,S12,S16,S17\right)$ of different methods for area A5 (***A***) and area A6 (***B***). The results of the fully affine invariant group and the feature-based group are represented by dashed lines and dotted lines, respectively. The solid lines show the correlation coefficient of unregistered ROIs mask-pair $\left\{ROIs_{Mask}{\;}_{n}^{common},ROIs_{Mask}{\;}_{template}^{common}\right\}$.

Figure 3 shows the correlation between $ROIs_{Mask}\overset{common}{\underset{template}{}}$ and the registered ${ROIs_{Mask}}_{n}^{common}$ for A5 and A6. *n* ∈ (*S*08, *S*09, *S*10, *S*11, *S*12, *S*16, *S*17). Figure 3*A* shows that fully affine invariant methods can reliably register FOV images across multiple sessions. In contrast, SIFT and BRISK failed to register the S16 and S17 of A5. Moreover, ORB failed to register the S10 and S11 of A5. Taken together, feature-based methods could easily fail when they cannot generate enough inliers. For area A6, the fully affine invariant group and feature-based group achieved similar results (Fig. 3*B*). In addition, Tables 1, 2 show that the mean ratios of inliers to initial matches (Inliers/Matched features) of the fully affine invariant group is lower than that of the feature-based group for both areas.

### Comparison between the fully affine invariant group and the conventional group

Different parameters were tested for methods in the conventional group to maximize their performance. The iteration of LK and ECC was set to 100, the number of levels for multiresolution was set to 3, and a total of four different transformation types (affine, translation, Euclidean, and homography) were compared on all FOV pairs. After comparison, Euclidean was applied because it produced the best results. For the non-rigid NoRMCorre method, five different square patch sizes (24, 32, 48, 96, 128) were tested with other parameters set as default values. Finally, the default patch size value 32 was adopted in the current study. For TurboReg, four different transformation types (affine, translation, rigid body, and bilinear) were examined on all FOV pairs. Lastly, a rigid body was employed in this study. For MOCO, the default parameters were used.

**Figure 4.**
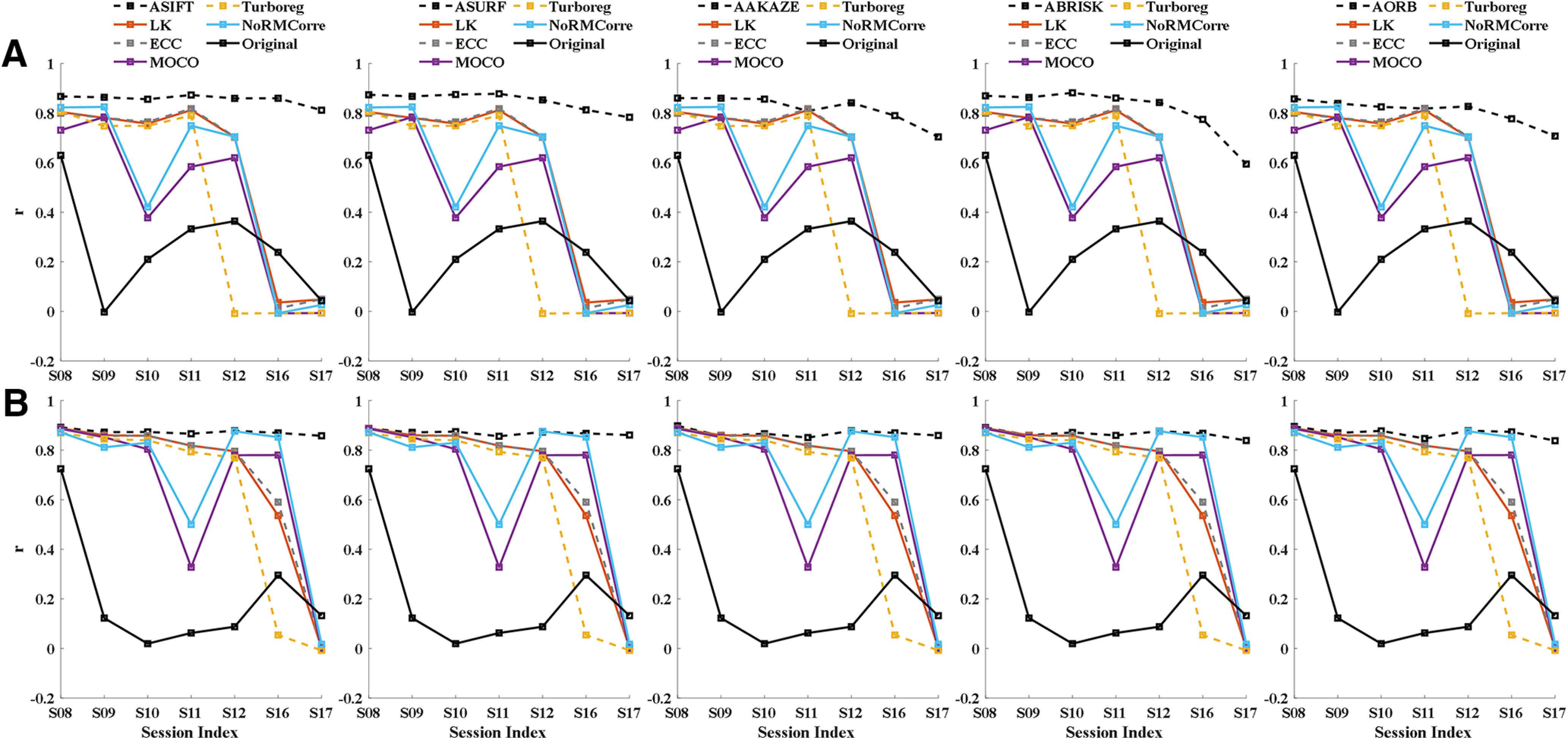
Comparison of performance between the fully affine invariant group and the conventional group. The correlation between $ROIs_{Mask}{\;}_{template}^{common}$ and the registered $ROIs_{Mask}{\;}_{n}^{common},n\in \left(S08,S09,S10,S11,S12,S16,S17\right)$ of different methods for area A5 (***A***) and A6 (***B***). The correlation coefficients of the fully affine invariant group and the unregistered pairs of $\left\{ROIs_{Mask}{\;}_{n}^{common},ROIs_{Mask}{\;}_{template}^{common}\right\}$ are represented by black dashed lines and black solid lines, respectively. The results of LK, ECC, MOCO, TurboReg, and NoRMCorre were shown in dark red, dashed gray, purple, dashed yellow, and blue color, respectively.

In Figure 4, we compared each method in the fully affine invariant group with all methods in the conventional group. As can be seen, ASIFT, ASURF, AAKAZE, ABRISK, and AORB outperformed the methods in the conventional group for most sessions from A5 and A6. For A5, all methods in the conventional group failed to register sessions that had low sharpness metrics (S17) or few common neurons (S16; Fig. 4*A*). For A6, the intensity-based methods, i.e., LK, ECC, and TurboReg, failed when the session had both a low sharpness metrics and a small common neuron number (S17). Moreover, the performance of MOCO and non-rigid NoRMCorre decreased in sessions with fewer common neurons (S11 and S17; Fig. 4*B*).

### Comparison between the fully affine invariant group and the CLAHE-based conventional group

**Figure 5.**
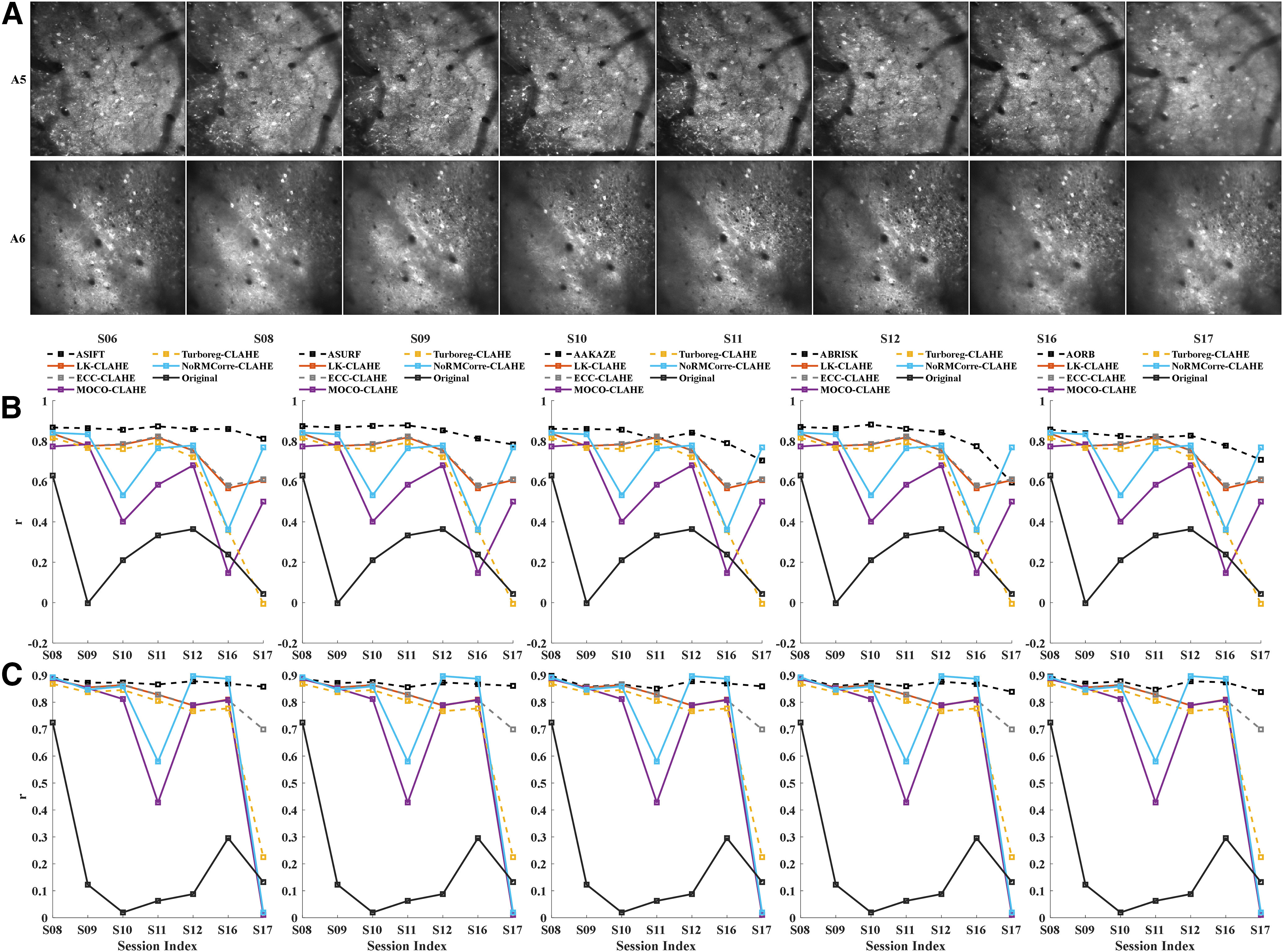
Comparison of performance between the fully affine invariant group and the CLAHE-based conventional group. ***A***, The CLAHE adjusted FOV image of each session from A5 (upper row) and A6 (lower row). CLAHE, contrast limited adaptive histogram equalization. The correlation between $ROIs_{Mask}{\;}_{template}^{common}$ and the registered $ROIs_{Mask}{\;}_{n}^{common},n\in \left(S08,S09,S10,S11,S12,S16,S17\right)$ of different methods for area A5 (***B***) and A6 (***C***). The correlation coefficients of the fully affine invariant group and the unregistered pairs of $\left\{ROIs_{Mask}{\;}_{n}^{common},ROIs_{Mask}{\;}_{template}^{common}\right\}$ are represented by black dashed lines and black solid lines, respectively. The results of LK-CLAHE, ECC-CLAHE, MOCO-CLAHE, TurboReg-CLAHE, and NoRMCorre-CLAHE were shown in dark red, dashed gray, purple, dashed yellow, and blue color, respectively.

We tested different parameters for CLAHE, and finally a block size of 8 × 8 and contrast limiting threshold = 1 were adopted in this study. Figure 5*A* shows the CLAHE adjusted FOV image of each session from A5 and A6. As can be seen, the local details in the images are more recognizable when compared with the results shown in Figure 2*A*,*C*.

**Figure 6.**
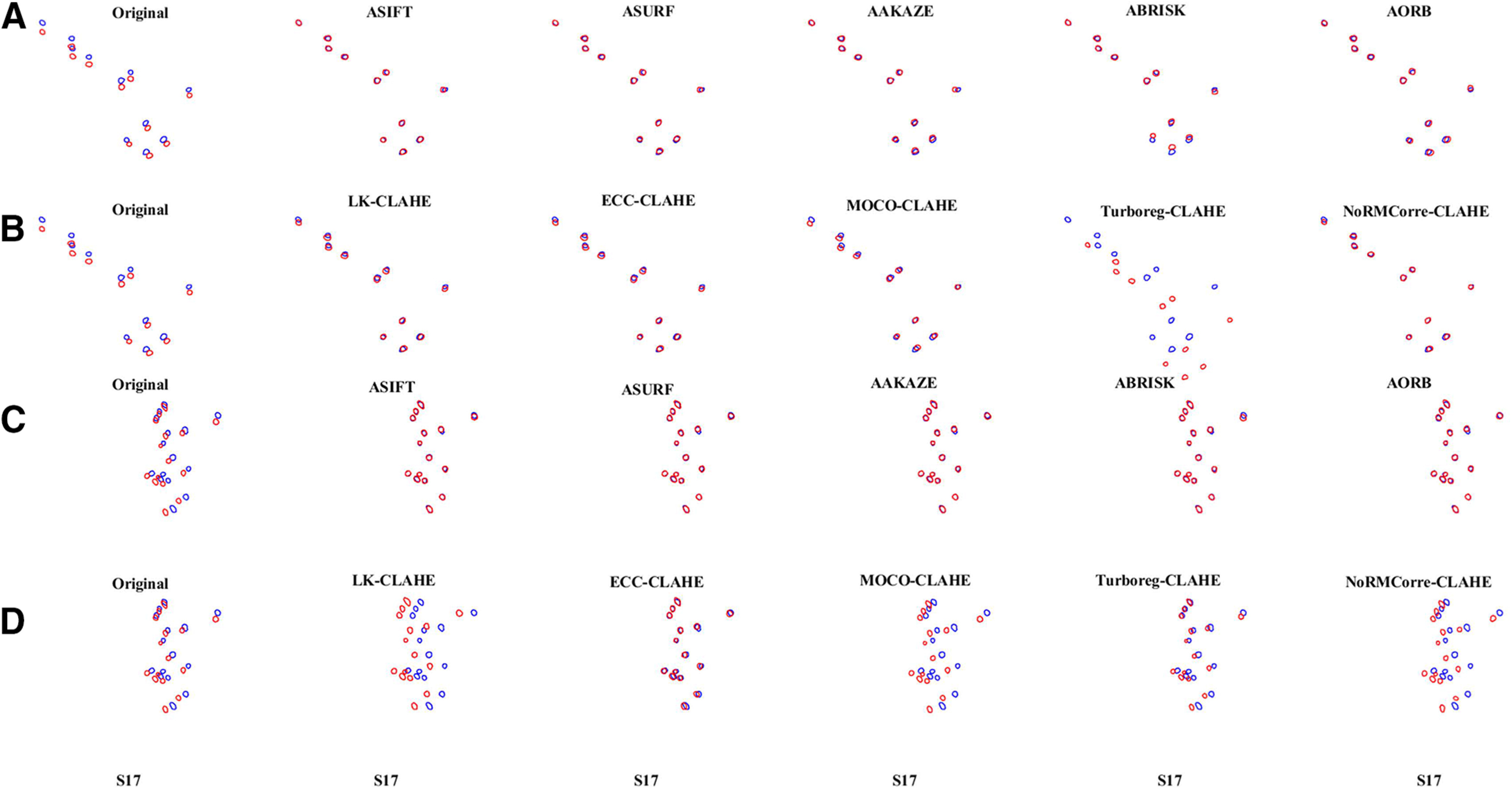
Visualization of the overlay of the ROIs mask-pairs on S17 of A5 and A6. The overlay of the $ROIs_{Mask}{\;}_{template}^{common}$ (blue) and the registered $ROIs_{Mask}{\;}_{n}^{common}$ (red) on S17 of the fully affine invariant group (***A***) and the CLAHE-based conventional group (***B***) for area A5. The overlay of the ${ROIs_{Mask}}_{template}^{common}$ (blue) and the registered $ROIs_{Mask}{\;}_{n}^{common}$ (red) on S17 of the fully affine invariant group (***C***) and the CLAHE-based conventional group (***D***) for area A6. The higher degree of overlap between the template and the registered ROIs mask, the better the alignment results.

Fully affine invariant methods were compared with CLAHE-based conventional methods (Fig. 5*B*,*C*). Results showed that ASIFT, ASURF, AAKAZE, ABRISK, and AORB outperformed the methods in the CLAHE-based conventional group for most of sessions from both A5 and A6. The performance of MOCO-CLAHE and non-rigid NoRMCorre-CLAHE decreased in sessions with small common neuron number (S16 for A5; S11 and S17 for A6). Besides, TurboReg-CLAHE failed to register the low sharpness session S17 for A5 and A6. Figure 6 visualizes the alignment results on S17 of the fully affine invariant group and the CLAHE-based conventional group for A5 (Fig. 6*A*,*B*) and A6 (Fig. 6*C*,*D*), respectively. The higher degree of overlap between the template and the registered ROIs mask, the better the alignment results. The overlap results are in line with the results shown in Figure 5*B*,*C*.

### The mean and standard error of alignment results for the four groups

**Figure 7.**
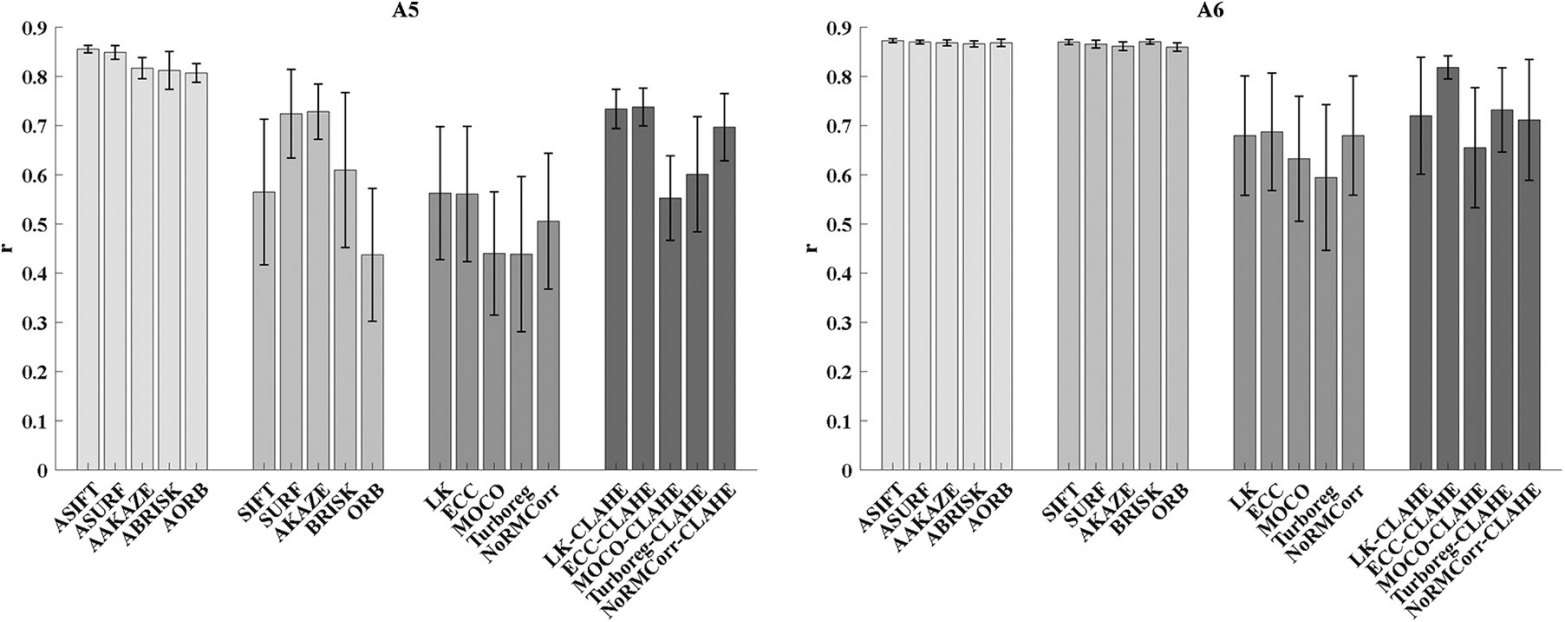
The mean ± SEM of the correlations on all registered ROIs mask-pairs $\left\{ROIs_{Mask}{\;}_{n}^{common},ROIs_{Mask}{\;}_{template}^{common}\right\}$ of area A5 (left) and A6 (right) for the four groups of approach.

The mean ± SEM of the correlation on all registered pairs $\left\{{ROIs_{Mask}}_{n}^{common},{ROIs_{Mask}}_{template}^{common}\right\}$ of A5 and A6 for the four groups of approach are shown in Figure 7. For A5, the results indicate that the fully affine invariant group achieves better results than all other groups. Moreover, the fully affine invariant group outperforms the conventional group and the CLAHE-based conventional group for A6. Besides, methods in the CLAHE-based conventional group outperform the corresponding methods in conventional group for both areas.

## Discussion

In this study, we introduce new methodologies for cross-session FOV alignment. We explore the performance of ASIFT, ASURF, AAKAZE, ABRISK, and AORB on FOV alignment of *in vivo* calcium imaging data, and we improve their reliability by using L1-norm. Furthermore, we compare their performance with general feature-based methods (SIFT, SURF, AKAZE, BRISK, ORB), the conventional methods (LK, ECC, MOCO, TurboReg, NoRMCorre), and the CLAHE-based conventional methods (LK-CLAHE, ECC-CLAHE, MOCO-CLAHE, TurboReg-CLAHE, NoRMCorre-CLAHE). Our results show that the fully affine invariant methods outperform the other methods in general and also in the case of image blurring, insufficient common neurons, and uneven background brightness. To the best of our knowledge, this is the first study that proves the feasibility of fully affine invariant methods in cross-session calcium FOV alignment. These methods could be useful for neuroscience research, especially for studies involving experience-dependent plasticity spanning over days or weeks.

Fully affine invariant methods outperform feature-based methods because they use different ways to extract keypoints. Specifically, feature-based methods only detect keypoints on original image pair, while fully affine-invariant methods also detect keypoints on simulated images caused by change of the viewing angle. However, the potential drawback is that full affine-invariant methods are more prone to accumulation of keypoints that are not discriminative. As a result, it could be difficult for NNDR and RANSAC to match keypoints as well as to keep inliers when the discrimination is low. Studies have shown that when the ratio of inliers to initial matches is low, methods like RANSAC are unlikely to find a good solution since it does not test enough hypotheses (Raguram et al., 2008). Moreover, RANSAC could produce different results each time because of its randomness (Hast et al., 2013). We tried to solve this problem by replacing RANSAC with other advanced methods, i.e., progressive sample consensus (PROSAC; Chum and Matas, 2005) and grid-based motion statistics (Bian et al., 2017). However, both methods cannot generate reproducible results when the ratio of inliers to initial matches is low (data not shown). In this study, we overcome this problem by repetitively running the NNDR and RANSAC for multiple times (100 times in the current study) and choosing the transformation matrix which minimizes L1-norm of the intensity difference where neuron appears between $\left\{{ROIs_{Mask}}_{n}^{raw},{ROIs_{Mask}}_{template}^{raw}\right\}$. After using this simple operation, fully affine invariant methods can achieve reproducible results even if they have a low inlier ratio.

Methods in the conventional group decrease their performance in sessions with small common neuron number for various reasons. Intensity-based methods, i.e., LK, ECC, and TurboReg, register images based on global intensity information. When the common neuron number is small, the intensity difference of the non-common area may have a larger impact on the results of registration than that of the common area, making it difficult to find the optimal solution. MOCO and non-rigid NoRMCorre register the image pair using frequency domain information within the whole image or single patch. MOCO cannot correct rotation artifacts which frequently happen in cross-session imaging. Non-rigid NoRMCorre may not be applicable when the patch does not contain enough spatial features (Mitani and Komiyama, 2018). In contrast, the fully affine invariant group registers the image pair using local keypoints as a statistic of the image content. They avoid to use global image content, thereby decreasing the negative effects of the different contents in the FOV image pair. Therefore, the fully affine invariant group outperforms the conventional group in the case of insufficient common neurons. Additionally, CLAHE increases the accuracy of methods in the conventional group because it improves image characteristics of uneven brightness regions. CLAHE enhances local image details by directly manipulating the intensity values of individual pixels in each image block. However, the results of methods in CLAHE-based conventional group are still inferior to those of fully affine invariant methods in most sessions. In other words, fully affine invariant methods do not require CLAHE to obtain reliable results. Thus, fully affine invariant methods are robust to uneven background brightness.

In this study, we improve the reliability of the fully affine invariant group by using an L1-norm. However, an alternative could be to use dimension reduction methods, such as principal components analysis, to increase the discrimination of keypoints. Besides, here, fully affine invariant methods are applied as offline methods for FOV alignment. It would be desirable to extend them as online registration methods, which will help the experimenter to more efficiently collect the same group of neurons across days or weeks in the experiments. Moreover, we did not include SIMA (Kaifosh et al., 2014) and Suite2p (Pachitariu et al., 2017) in our study, because alignment methods adopted by SIMA and Suite2p are not designed for multiday recording. However, we compared our proposed methods with the built-in method of CaImAn, i.e., NoRMCorre.

This study is the first and comprehensive work investigating the performance of ASIFT, ASURF, AAKAZE, ABRISK, and AORB on longitudinal cellular resolution calcium imaging data. These methods will be useful for many neuroscience studies involving chronic changes in neuronal activities. Moreover, although ASIFT, ASURF, AAKAZE, ABRISK, and AORB are conducted on two-photon microscopy-based calcium imaging data, these methods should be promising in registering FOV images collected by one-photon or widefield fluorescence microscopy.

## Synthesis

Reviewing Editor: Zoltan Nusser, Institute of Experimental Medicine Decisions are customarily a result of the Reviewing Editor and the peer reviewers coming together and discussing their recommendations until a consensus is reached. When revisions are invited, a fact-based synthesis statement explaining their decision and outlining what is needed to prepare a revision will be listed below. The following reviewer(s) agreed to reveal their identity: NONE.

In this manuscript, the authors evaluated many different algorithms for aligning fields of view across calcium imaging experiments. They found one class of algorithms with significantly better robustness compared to the others, and further improved on these algorithms with some simple tweaks. This is a very useful method and it should be published when the authors address the concerns raised by the reviewers.

Comments:

1. The measure of performance used by the authors is the normalized cross-correlation coefficient between ROI masks, but there is no mention in the Methods as to how this is calculated. Is it really the cross-correlation or simply the correlation of the masks? Please provide more information. I also have questions about the limitations of this coefficient: the cross-correlation should be two-dimensional to account for small offsets of the masks, but is the coefficient taken at the ‘zero-lag’ or is it the maximum coefficient calculated within a reasonable range of displacements/offset (for example, 50 pixels maximum offset for the 2D-cross correlation)? I am particularly curious to know how the authors get a r value of 0 or nearly 0 (figure 5 and 6). When I look at the raw images, they don’t look so different.

2. Although this r value is a valid quantification to evaluate the goodness of the transformation, a more straightforward and informative measure would simply be the correlation coefficient between a template image and a transformed image (image as in the motion-corrected, time-averaged FOV). Can the authors provide this measure as well?

3. What is the rationale for taking the first imaged session as the template? Shouldn’t the template be defined as the session with the highest average coefficient to all other sessions? The coefficients reported in Figure 5 and 6 are already high (>0.8 or 0.9), but a suggestion to even improve these values would be to successively use every session as potential template, then run the transformations on all the other sessions, and finally keep the session with the highest performance as the template.

4. Table 3 is an incredible resource that summarizes most of the results presented throughout the manuscript. I would strongly suggest to convert this table into a figure with bars and error bars, showing the mean and s.e.m for both A5 and A6. This figure could serve as a summary figure for the readers with proper statistical analysis.

5. “Neurons with ambiguous 130 boundary were excluded in this study.” What do the authors consider “ambiguous” and is there a more systematic way to quantify this ambiguity (number of pixels, circularity, etc, ...)? One could imagine that some detected ROIs were manually discarded because poorly transformed, and therefore only the good ROIs were kept, leading to the high r values shown in the Figures.

6. The codes provided by the authors are written half in Python and half in Matlab. My suggestion for the authors is to convert their codes into one format for the sake of portability. I would encourage Python for its open source access.

7. My last point is about the figures in general. I would encourage the authors to merge some figures together for the sake of readability. My suggestions are as follow:

a. Figure 1 and 2 can be merged together to depict the entire ‘pipeline’, from data acquisition to image transformations.

b. Figure 3 and 4 can be merged to show the raw images together with their characteristics without having to scroll between two different figures to have access to this information.

c. It would also make sense to merge Figure 7 and 8.

d. Finally Figure 9 and 10 could be merged together and only show a selected number of ROIs masks (the best and worst for each category, for example).

e. See comment #4 if you decide to include this figure.

Finally, I would encourage the authors to have a thorough look at the manuscript and correct obvious grammatical errors (i.e. non-overlapped blocks -> non-overlapping blocks; laptop, which equipped with an Intel -> laptop equipped with an Intel, etc...).

My main concern is about the release of the code. Right now, I think all of the code for ASIFT&co is inside the demo jupyter notebooks. This code should be made into a proper package with functions that the user can call, which have well explained options and inputs. The package should be deposited on PyPI. That package will be the main contribution of this paper, and the only thing that needs to be revised for resubmission. The demo scripts also should be revised to call the functions from the corresponding package. If I had to review the useability of the code right now, it would get very poor marks...

## Author Response

Manuscript Instructions: The sex of the species studied is not mentioned in the materials and methods section. Please mention “male", “female", “x males and x females", “of either sex”.

RESPONSE: The sex of the mouse has now been added in the materials and methods section. Please see line 106 on page 5.

Comment #1: “The measure of performance used by the authors is the normalized cross-correlation coefficient between ROI masks, but there is no mention in the Methods as to how this is calculated. Is it really the cross-correlation or simply the correlation of the masks? Please provide more information. I also have questions about the limitations of this coefficient: the cross-correlation should be two-dimensional to account for small offsets of the masks, but is the coefficient taken at the ‘zero-lag’ or is it the maximum coefficient calculated within a reasonable range of displacements/offset (for example, 50 pixels maximum offset for the 2D-cross correlation)?"

RESPONSE: Thank you very much for pointing out our mistake in the wording. We indeed used the correlation of the ROI masks as the measure of performance. Specifically, we first reshape the 2D ROI masks into 1D vectors, then calculate their Pearson’s linear correlation coefficient. The ROI masks are binary images containing values only 0 or 255. Therefore, the higher the correlation coefficient, the more similar the masks. We have corrected this mistake in various places in the revised manuscript (please see line 176-181 on Page 9, line 255 on Page 12, line 304 on Page 15, line 509, 515 and 523 on Page 25).

"I am particularly curious to know how the authors get a r value of 0 or nearly 0 (figure 5 and 6). When I look at the raw images, they don’t look so different.”

RESPONSE: Thanks a lot for pointing out this point of confusion. The r values shown in Fig. 5 and Fig. 6 represent the similarity of the raw unregistered pairs of {ROIs_(Mask_n )^Common,ROIs_(Mask_Template )^Common } of A5 and A6. The low r value is due to the small overlap between the unregistered pairs of {ROIs_(Mask_n )^Common,ROIs_(Mask_Template )^Common } (shown in Figure S1). Again, thank you very much for your careful reading.

Figure S1. Visualization of the overlay of the raw unregistered pairs of {ROIs_(Mask_n )^Common,ROIs_(Mask_Template )^Common } of A5 and A6. The template is the first imaged session (S06) of A5 and A6. The circles represent the contours of the neurons common to the unregistered pairs of {ROIs_(Mask_n )^Common,ROIs_(Mask_Template )^Common }. The blue color labels the contours of the neurons in S06. The red color labels the contours of the neurons in other sessions.

Comment #2: “Although this r value is a valid quantification to evaluate the goodness of the transformation, a more straightforward and informative measure would simply be the correlation coefficient between a template image and a transformed image (image as in the motion-corrected, time-averaged FOV). Can the authors provide this measure as well?"

RESONSE: This is a very good point. If the correlation coefficient between a template FOV and a transformed FOV could correctly evaluate the performance of alignment methods, then it would be a more straightforward measure. However, we found that it cannot properly estimate the performance of the method TurboReg. Take the FOV image of session17 (S17) from A5 as an example (Figure S2 shown below). For TurboReg, the correlation of the common area (contents within the yellow rectangle) between the template FOV of S06 and the transformed FOV image of S17 is 0.6743. While the correlation of the registered pairs of {ROIs_(Mask_S17 )^Common,ROIs_(Mask_Template )^Common } is -0.0059. The possible reason is that TurboReg attempts to minimize the squared difference in intensity value between the template and the registered image, which results in a high correlation coefficient between FOV images but may not generate the optimal alignment result. In contrast, for ASIFT, the correlation of the common area between the FOV image of S06 and the registered FOV of S17 is 0.6355, and the correlation of the registered pairs of {ROIs_(Mask_S17 )^Common,ROIs_(Mask_Template )^Common } is 0.8114. Therefore, the correlation of the registered pairs of {ROIs_(Mask_n )^Common,ROIs_(Mask_Template )^Common } is a more valid evaluation metric than the correlation of the FOV images.

Figure S2. The alignment results and the overlay of the (ROIs)_((Mask)_Template )^Common (white) and the registered ROIs_(Mask_S17 )^Common (yellow) of S17 for method TurboReg and ASIFT.

Comment #3: “What is the rationale for taking the first imaged session as the template? Shouldn’t the template be defined as the session with the highest average coefficient to all other sessions? The coefficients reported in Figure 5 and 6 are already high (>0.8 or 0.9), but a suggestion to even improve these values would be to successively use every session as potential template, then run the transformations on all the other sessions, and finally keep the session with the highest performance as the template.”

RESONSE: We thank the reviewer for the stimulating questions and useful suggestions. For chronic studies that involve experience-dependent modification of neural activities, we typically are interested in the dynamics of the same group of neurons from beginning to end. So, we usually take the neurons appearing in the first imaged session as the target neurons and track their neural activity throughout the whole experimental period. Besides, for long-term recordings, the quality of the microscopic image may gradually decrease due to photobleaching, phototoxicity, and brain state change. Thus, the first imaged session usually has the best signal-to-noise ratio. Taking your suggestions into consideration, we have now added an option in our code, which allows users to select the template according to their needs or experimental conditions. The default value of this option is taking the first imaged session as the template. However, users can change this value to decide which session, i.e. template, to align the FOV images.

Comment #4: “Table 3 is an incredible resource that summarizes most of the results presented throughout the manuscript. I would strongly suggest converting this table into a figure with bars and error bars, showing the mean and s.e.m for both A5 and A6. This figure could serve as a summary figure for the readers with proper statistical analysis.”

RESPONSE: Thank you for the positive comments and useful suggestions. As advised, Table 3 has now been converted to a bar graph with mean and s.e.m (please see Figure 7, line 303 and 304 on Page 15, line 536 on Page 26).

Comment #5: “‘Neurons with ambiguous boundary were excluded in this study.’ What do the authors consider ‘ambiguous’ and is there a more systematic way to quantify this ambiguity (number of pixels, circularity, etc, ...)? One could imagine that some detected ROIs were manually discarded because poorly transformed, and therefore only the good ROIs were kept, leading to the high r values shown in the Figures.”

RESPONSE: We thank the reviewer for this valid and important comment. To better address this issue and to improve the portability of our code, we replaced the Fiji-based Trainable Weka Segmentation (TWS) method with the Python-based Cellpose (Stringer C et al., 2020) method. Compared with TWS, Cellpose can precisely segment neurons of various types and without the needs of model retraining or parameter adjustments. Since dendritic spines can easily be misdetected as neurons, we excluded them by requiring that the ROI mask of individual neurons should contain at least 60 pixels. The number of ROIs detected by TWS and Cellpose are different (Figure S3 shown below). However, this did not affect the alignment results of fully affine invariant methods, i.e. ASIFT, ASURF, AAKAZE, ABRISK, and AORB. We calculated the average of r values obtained on different sessions and found that the change of the average of different methods is less than 0.05 (Figure S4 shown below).

We have now replaced the TWS method and with the following description: “Cellpose (Stringer C et al., 2020) was applied to the mean calcium image of each session to get the corresponding ROIs mask. Cellpose can precisely segment neurons of various types and without the needs of model retraining or parameter adjustments. Since dendritic spines can easily be misdetected as neurons, we excluded them by requiring that the ROI mask of individual neurons should contain at least 60 pixels.” Please see line 120-125 of Page 6.

Figure S3. The number of ROIs detected by TWS and Cellpose methods for different sessions from the area A5 (left) and the area A6 (right).

Figure S4. The average of r values of different methods obtained on different sessions from the area A5 (left) and the area A6 (right).

Besides, we have added a simple strategy to improve the performance of the feature-based methods, i.e. SIFT, SURF, AKAZE, BRISK, and ORB. Specifically, the feature-based methods would generate distorted FOV images if some important inliers are missing. We detected the contours of the registered image. If their contour number is less than half the number of the original images, we treat the registered image as distorted result and exclude it. Please see the new results in figure 3. The corresponding description are also modified, please see line 251-254 and line 258 on Page 12, line 259 on Page 13.

Comment #6: “The codes provided by the authors are written half in Python and half in Matlab. My suggestion for the authors is to convert their codes into one format for the sake of portability. I would encourage Python for its open source access.”

RESPONSE: Thank you very much for your practical advice. We have now produced a python-based package, which will be deposited on PyPI after revision. The usage of this package has been described in the README file within the folder of “FAIM_package”. The package includes all fully affine invariant methods. The FOV images used in the manuscript were included in a folder named “examples”. We did not include methods of the control groups in this package because some of them (MOCO, LK, ECC, and NoRMCorr) were written in MATLAB or Fiji. However, we uploaded another folder named “AffineCa2p_reproduce_results” in the resubmission containing all the methods for verification of our results, which we plan to upload to GitHub after the revision process. For convenience of use, we have simplified the codes. The processing procedure was reduced from 11 steps to 2 steps. We hope this serves the purpose. The usage of this code has been described in the README file within the folder of “AffineCa2p_reproduce_results”.

Comment #7: “My last point is about the figures in general. I would encourage the authors to merge some figures together for the sake of readability. My suggestions are as follow:

a. Figure 1 and 2 can be merged together to depict the entire ‘pipeline’, from data acquisition to image transformations.

b. Figure 3 and 4 can be merged to show the raw images together with their characteristics without having to scroll between two different figures to have access to this information.

c. It would also make sense to merge Figure 7 and 8.

d. Finally Figure 9 and 10 could be merged together and only show a selected number of ROIs masks (the best and worst for each category, for example).

e. See comment #4 if you decide to include this figure.”

RESPONSE: We appreciate your comprehensive reviewing and constructive suggestions, which helped a lot to improve the structure of our manuscript. The figures and the corresponding legends have been modified according to the suggestions (please see line 143 on Page 7, line 161 on Page 8, line 182 on Page 9, line 232 and line 234 on Page 11, line 240, 242, 255 and 256 on Page 12, line 261, 276, and 280 on Page 13, line 284, 288, 290, 291, 293, 298, 299, 300, 302 on Page 14, line 306 on Page 15, line 480-484, 487, 493, 500, 504, 505 on Page 24, line 506, 507, 508, 514, 521-522 on Page 25, line 530, 532, 533, 534, 536, 537 on Page 26).

Comment #8: “Finally, I would encourage the authors to have a thorough look at the manuscript and correct obvious grammatical errors (i.e. non-overlapped blocks -> non-overlapping blocks; laptop, which equipped with an Intel -> laptop equipped with an Intel, etc...).”

RESPONSE: Many thanks for the very careful reading and useful suggestions. These errors have now been corrected in the revised manuscript (please see line 198-199 on Page 10 and line 221 on Page 11). The corrections are shown in our revised manuscript with blue color.

Comment #9: “My main concern is about the release of the code. Right now, I think all of the code for ASIFT&co is inside the demo jupyter notebooks. This code should be made into a proper package with functions that the user can call, which have well explained options and inputs. The package should be deposited on PyPI. That package will be the main contribution of this paper, and the only thing that needs to be revised for resubmission. The demo scripts also should be revised to call the functions from the corresponding package. If I had to review the useability of the code right now, it would get very poor marks.”

RESPONSE: Again, we thank for the suggestions. The codes have been made into a package and which will be deposited on PyPI after the revision process. The README file has been included in the folder of “FAIM_package”. For further detail, users can use the fully affine invariant methods by calling a function named “FAIMCaSig.AlignIm”. We also provide users with 6 well explained options to input images through (1) path of the folder containing FOV images; (2) whether to provide the path of ROI masks; if the value is empty, the code will automatically extract ROI masks using cellpose; (3) whether to use contrast adjustment for the original FOV images; (4) which FOV image to use as a template; (5) the number of iterations of fully affine invariant method; and to decide (6) which fully affine invariant method to use. The function outputs the transformation matrix, the registered image, as well as the overlay of the ROI masks. However, methods in control groups are not included in this package because some of them (the MOCO, LK, ECC, and NoRMCorr) are written in MATLAB or Fiji. We uploaded another folder named “AffineCa2p_reproduce_results” containing all methods for reproducing the complete results. As mentioned, after the revision process is complete, this source file will be uploaded to GitHub. The usage of this code has been described in the README file within the folder of “AffineCa2p_reproduce_results"
