# Supplementary figures and images for: Fully Affine Invariant Methods for Cross-Session Registration of Calcium Imaging Data
Source: eNeuro. 2020 Aug 7;7(4):ENEURO.0054-20.2020. doi: 10.1523/ENEURO.0054-20.2020 (PMC7438060; doi:10.1523/ENEURO.0054-20.2020)

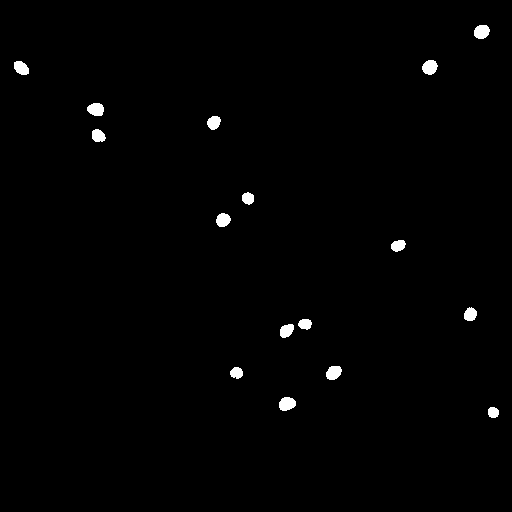

Supplement: Extended Data. — A zip file (named “data_code.zip”), including PyPI package (“FAIM_package” folder), example FOV images (within “examples” folder), and codes used to reproduce all results (within “AffineCa2p_reproduce_results” folder) were submitted as Extended Data. Each folder contains a readme file. Download Extended Data, EPS file. [file enu-eN-MNT-0054-20-s02.zip › data_code/AffineCa2p_reproduce_results/A5/raw_common_neurons/reg/ROI_day08_frame.tiff]

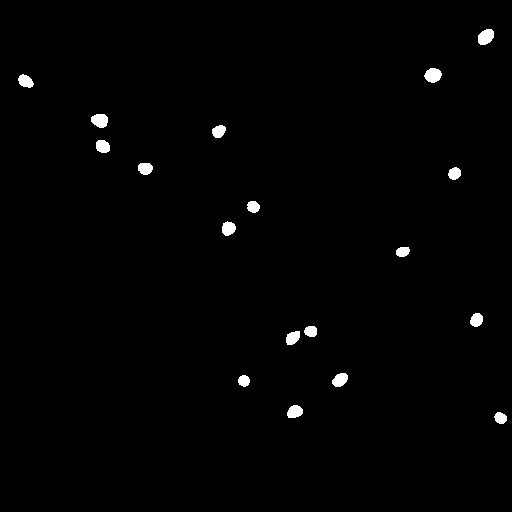

Supplement: Extended Data. — A zip file (named “data_code.zip”), including PyPI package (“FAIM_package” folder), example FOV images (within “examples” folder), and codes used to reproduce all results (within “AffineCa2p_reproduce_results” folder) were submitted as Extended Data. Each folder contains a readme file. Download Extended Data, EPS file. [file enu-eN-MNT-0054-20-s02.zip › data_code/AffineCa2p_reproduce_results/A5/raw_common_neurons/reg/ROI_day09_frame.tiff]

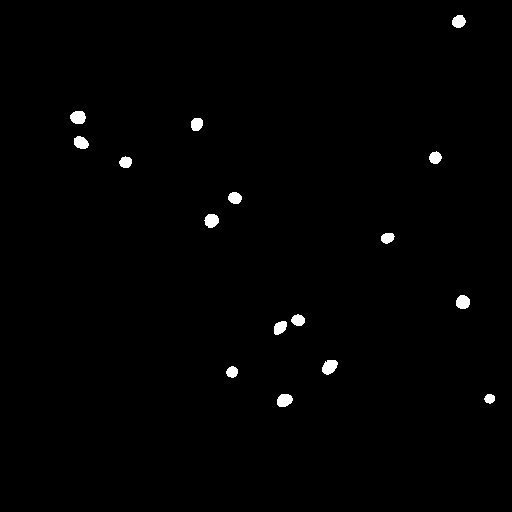

Supplement: Extended Data. — A zip file (named “data_code.zip”), including PyPI package (“FAIM_package” folder), example FOV images (within “examples” folder), and codes used to reproduce all results (within “AffineCa2p_reproduce_results” folder) were submitted as Extended Data. Each folder contains a readme file. Download Extended Data, EPS file. [file enu-eN-MNT-0054-20-s02.zip › data_code/AffineCa2p_reproduce_results/A5/raw_common_neurons/reg/ROI_day10_frame.tiff]

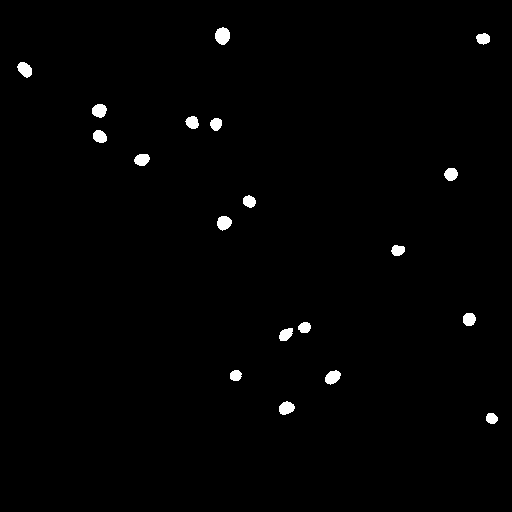

Supplement: Extended Data. — A zip file (named “data_code.zip”), including PyPI package (“FAIM_package” folder), example FOV images (within “examples” folder), and codes used to reproduce all results (within “AffineCa2p_reproduce_results” folder) were submitted as Extended Data. Each folder contains a readme file. Download Extended Data, EPS file. [file enu-eN-MNT-0054-20-s02.zip › data_code/AffineCa2p_reproduce_results/A5/raw_common_neurons/reg/ROI_day11_frame.tiff]

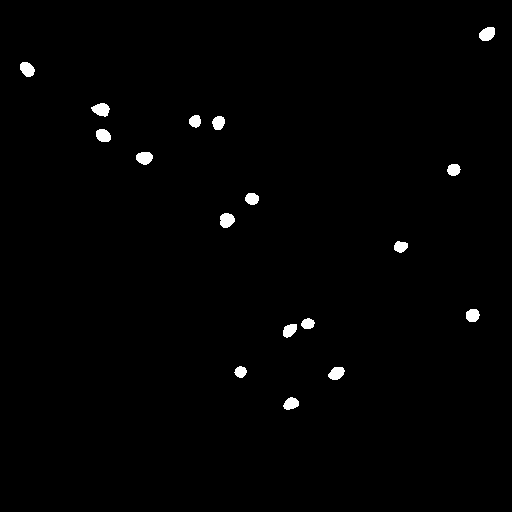

Supplement: Extended Data. — A zip file (named “data_code.zip”), including PyPI package (“FAIM_package” folder), example FOV images (within “examples” folder), and codes used to reproduce all results (within “AffineCa2p_reproduce_results” folder) were submitted as Extended Data. Each folder contains a readme file. Download Extended Data, EPS file. [file enu-eN-MNT-0054-20-s02.zip › data_code/AffineCa2p_reproduce_results/A5/raw_common_neurons/reg/ROI_day12_frame.tiff]

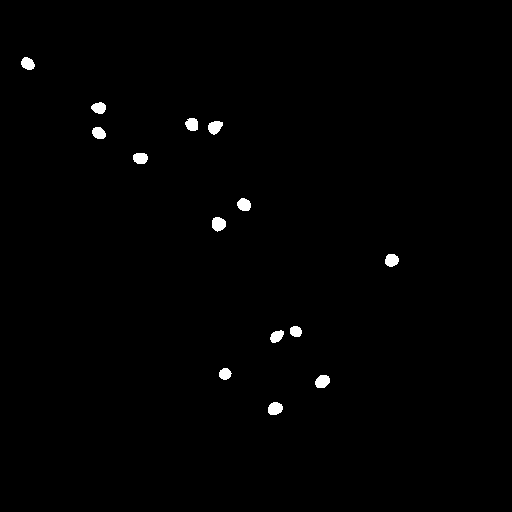

Supplement: Extended Data. — A zip file (named “data_code.zip”), including PyPI package (“FAIM_package” folder), example FOV images (within “examples” folder), and codes used to reproduce all results (within “AffineCa2p_reproduce_results” folder) were submitted as Extended Data. Each folder contains a readme file. Download Extended Data, EPS file. [file enu-eN-MNT-0054-20-s02.zip › data_code/AffineCa2p_reproduce_results/A5/raw_common_neurons/reg/ROI_day16_frame.tiff]

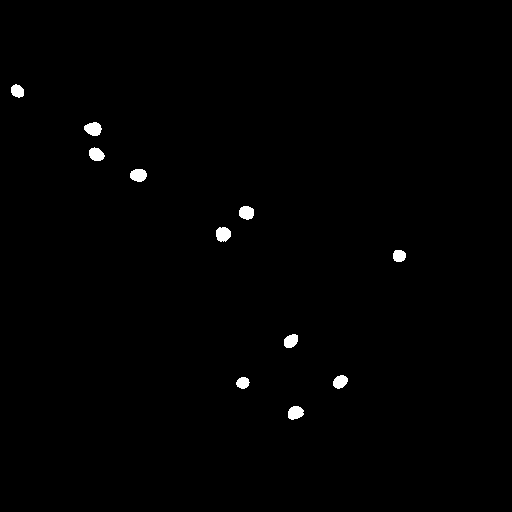

Supplement: Extended Data. — A zip file (named “data_code.zip”), including PyPI package (“FAIM_package” folder), example FOV images (within “examples” folder), and codes used to reproduce all results (within “AffineCa2p_reproduce_results” folder) were submitted as Extended Data. Each folder contains a readme file. Download Extended Data, EPS file. [file enu-eN-MNT-0054-20-s02.zip › data_code/AffineCa2p_reproduce_results/A5/raw_common_neurons/reg/ROI_day17_frame.tiff]

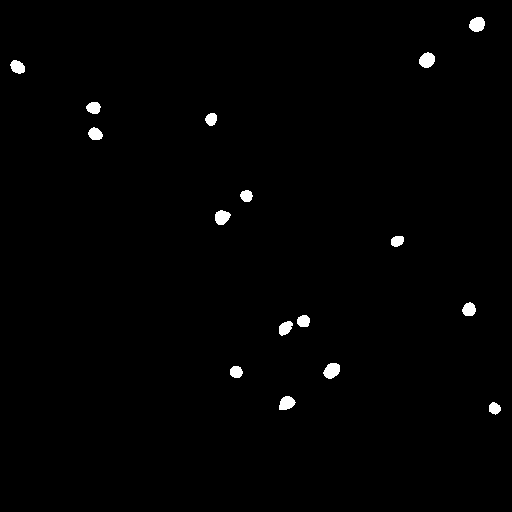

Supplement: Extended Data. — A zip file (named “data_code.zip”), including PyPI package (“FAIM_package” folder), example FOV images (within “examples” folder), and codes used to reproduce all results (within “AffineCa2p_reproduce_results” folder) were submitted as Extended Data. Each folder contains a readme file. Download Extended Data, EPS file. [file enu-eN-MNT-0054-20-s02.zip › data_code/AffineCa2p_reproduce_results/A5/raw_common_neurons/template/ROI_day06_08_frame.tiff]

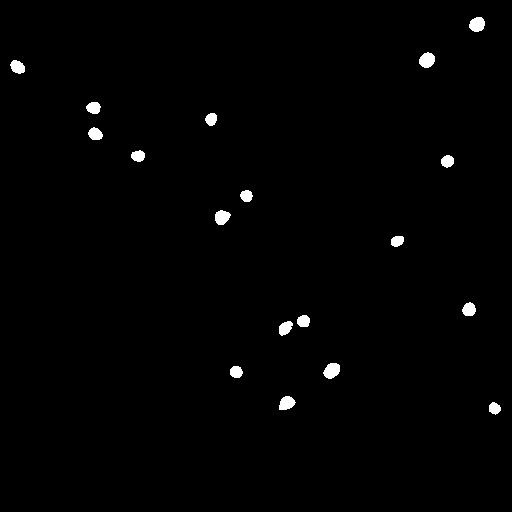

Supplement: Extended Data. — A zip file (named “data_code.zip”), including PyPI package (“FAIM_package” folder), example FOV images (within “examples” folder), and codes used to reproduce all results (within “AffineCa2p_reproduce_results” folder) were submitted as Extended Data. Each folder contains a readme file. Download Extended Data, EPS file. [file enu-eN-MNT-0054-20-s02.zip › data_code/AffineCa2p_reproduce_results/A5/raw_common_neurons/template/ROI_day06_09_frame.tiff]

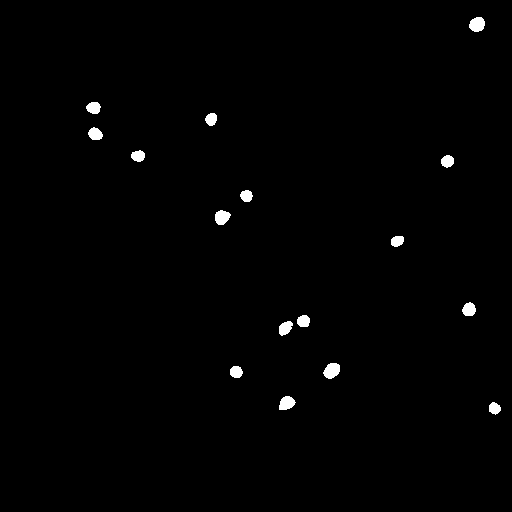

Supplement: Extended Data. — A zip file (named “data_code.zip”), including PyPI package (“FAIM_package” folder), example FOV images (within “examples” folder), and codes used to reproduce all results (within “AffineCa2p_reproduce_results” folder) were submitted as Extended Data. Each folder contains a readme file. Download Extended Data, EPS file. [file enu-eN-MNT-0054-20-s02.zip › data_code/AffineCa2p_reproduce_results/A5/raw_common_neurons/template/ROI_day06_10_frame.tiff]

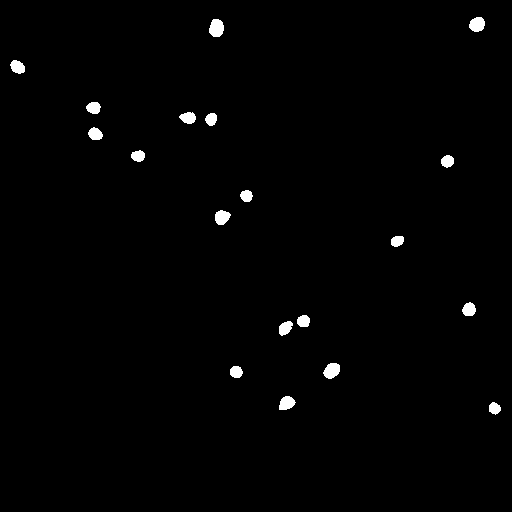

Supplement: Extended Data. — A zip file (named “data_code.zip”), including PyPI package (“FAIM_package” folder), example FOV images (within “examples” folder), and codes used to reproduce all results (within “AffineCa2p_reproduce_results” folder) were submitted as Extended Data. Each folder contains a readme file. Download Extended Data, EPS file. [file enu-eN-MNT-0054-20-s02.zip › data_code/AffineCa2p_reproduce_results/A5/raw_common_neurons/template/ROI_day06_11_frame.tiff]

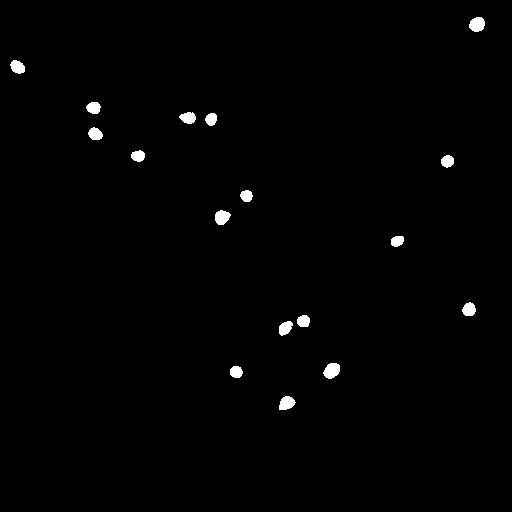

Supplement: Extended Data. — A zip file (named “data_code.zip”), including PyPI package (“FAIM_package” folder), example FOV images (within “examples” folder), and codes used to reproduce all results (within “AffineCa2p_reproduce_results” folder) were submitted as Extended Data. Each folder contains a readme file. Download Extended Data, EPS file. [file enu-eN-MNT-0054-20-s02.zip › data_code/AffineCa2p_reproduce_results/A5/raw_common_neurons/template/ROI_day06_12_frame.tiff]

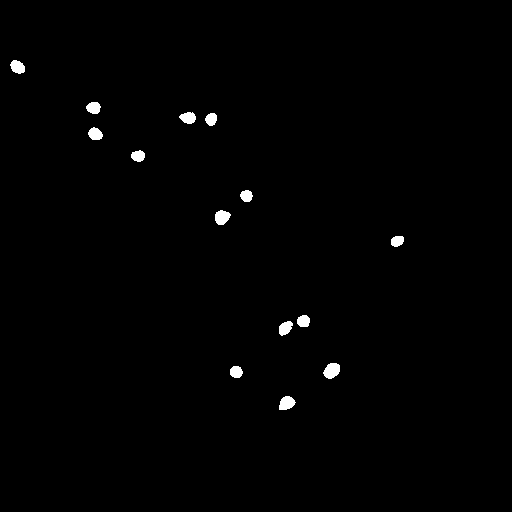

Supplement: Extended Data. — A zip file (named “data_code.zip”), including PyPI package (“FAIM_package” folder), example FOV images (within “examples” folder), and codes used to reproduce all results (within “AffineCa2p_reproduce_results” folder) were submitted as Extended Data. Each folder contains a readme file. Download Extended Data, EPS file. [file enu-eN-MNT-0054-20-s02.zip › data_code/AffineCa2p_reproduce_results/A5/raw_common_neurons/template/ROI_day06_16_frame.tiff]

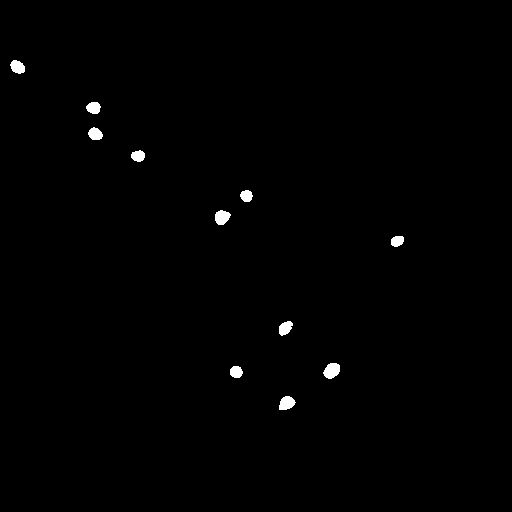

Supplement: Extended Data. — A zip file (named “data_code.zip”), including PyPI package (“FAIM_package” folder), example FOV images (within “examples” folder), and codes used to reproduce all results (within “AffineCa2p_reproduce_results” folder) were submitted as Extended Data. Each folder contains a readme file. Download Extended Data, EPS file. [file enu-eN-MNT-0054-20-s02.zip › data_code/AffineCa2p_reproduce_results/A5/raw_common_neurons/template/ROI_day06_17_frame.tiff]

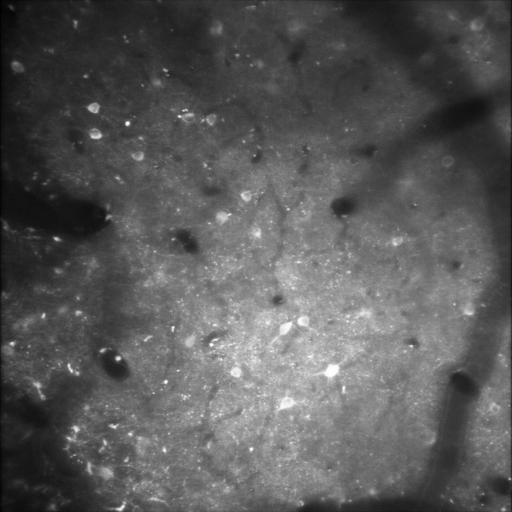

Supplement: Extended Data. — A zip file (named “data_code.zip”), including PyPI package (“FAIM_package” folder), example FOV images (within “examples” folder), and codes used to reproduce all results (within “AffineCa2p_reproduce_results” folder) were submitted as Extended Data. Each folder contains a readme file. Download Extended Data, EPS file. [file enu-eN-MNT-0054-20-s02.zip › data_code/AffineCa2p_reproduce_results/A5/reg_day06A5.png]

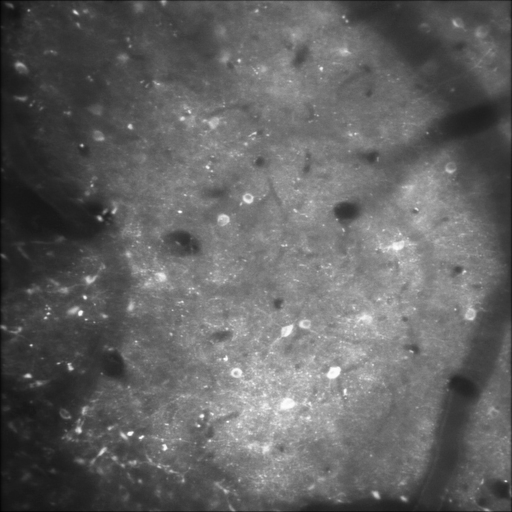

Supplement: Extended Data. — A zip file (named “data_code.zip”), including PyPI package (“FAIM_package” folder), example FOV images (within “examples” folder), and codes used to reproduce all results (within “AffineCa2p_reproduce_results” folder) were submitted as Extended Data. Each folder contains a readme file. Download Extended Data, EPS file. [file enu-eN-MNT-0054-20-s02.zip › data_code/AffineCa2p_reproduce_results/A5/reg_day08A5.png]

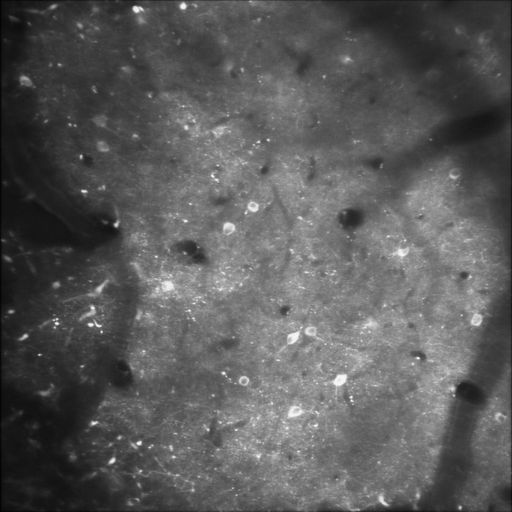

Supplement: Extended Data. — A zip file (named “data_code.zip”), including PyPI package (“FAIM_package” folder), example FOV images (within “examples” folder), and codes used to reproduce all results (within “AffineCa2p_reproduce_results” folder) were submitted as Extended Data. Each folder contains a readme file. Download Extended Data, EPS file. [file enu-eN-MNT-0054-20-s02.zip › data_code/AffineCa2p_reproduce_results/A5/reg_day09A5.png]

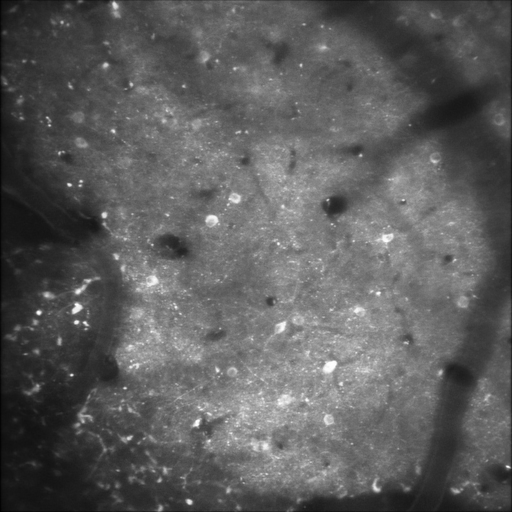

Supplement: Extended Data. — A zip file (named “data_code.zip”), including PyPI package (“FAIM_package” folder), example FOV images (within “examples” folder), and codes used to reproduce all results (within “AffineCa2p_reproduce_results” folder) were submitted as Extended Data. Each folder contains a readme file. Download Extended Data, EPS file. [file enu-eN-MNT-0054-20-s02.zip › data_code/AffineCa2p_reproduce_results/A5/reg_day10A5.png]

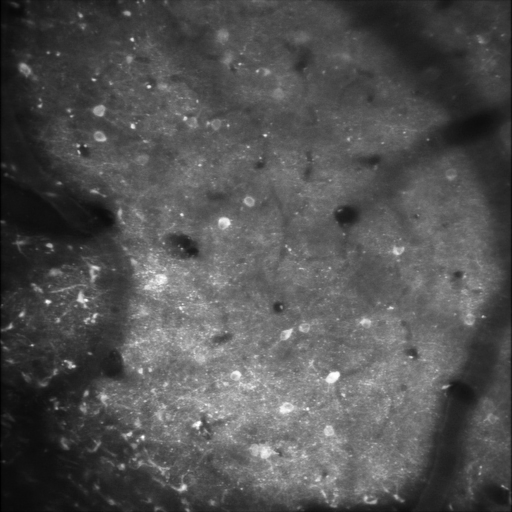

Supplement: Extended Data. — A zip file (named “data_code.zip”), including PyPI package (“FAIM_package” folder), example FOV images (within “examples” folder), and codes used to reproduce all results (within “AffineCa2p_reproduce_results” folder) were submitted as Extended Data. Each folder contains a readme file. Download Extended Data, EPS file. [file enu-eN-MNT-0054-20-s02.zip › data_code/AffineCa2p_reproduce_results/A5/reg_day11A5.png]

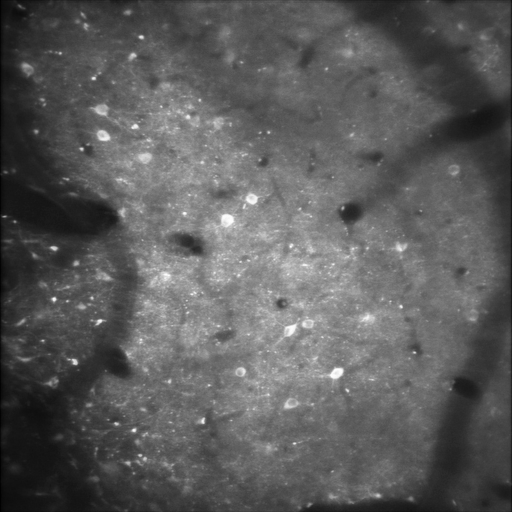

Supplement: Extended Data. — A zip file (named “data_code.zip”), including PyPI package (“FAIM_package” folder), example FOV images (within “examples” folder), and codes used to reproduce all results (within “AffineCa2p_reproduce_results” folder) were submitted as Extended Data. Each folder contains a readme file. Download Extended Data, EPS file. [file enu-eN-MNT-0054-20-s02.zip › data_code/AffineCa2p_reproduce_results/A5/reg_day12A5.png]

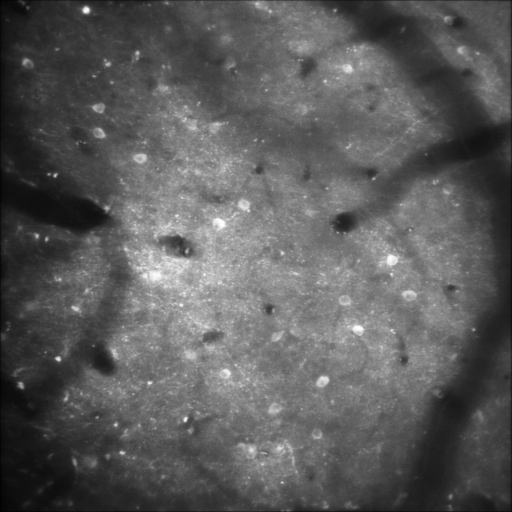

Supplement: Extended Data. — A zip file (named “data_code.zip”), including PyPI package (“FAIM_package” folder), example FOV images (within “examples” folder), and codes used to reproduce all results (within “AffineCa2p_reproduce_results” folder) were submitted as Extended Data. Each folder contains a readme file. Download Extended Data, EPS file. [file enu-eN-MNT-0054-20-s02.zip › data_code/AffineCa2p_reproduce_results/A5/reg_day16A5.png]

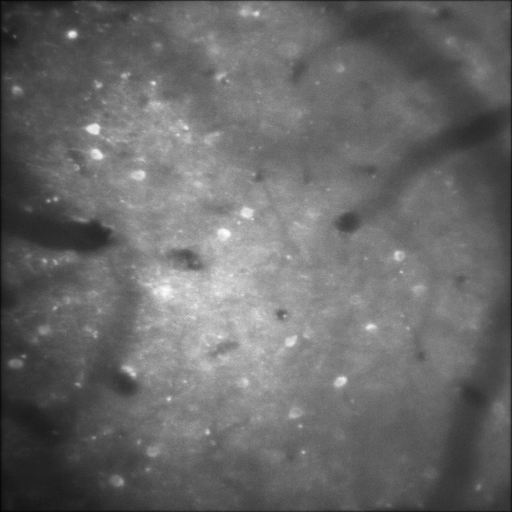

Supplement: Extended Data. — A zip file (named “data_code.zip”), including PyPI package (“FAIM_package” folder), example FOV images (within “examples” folder), and codes used to reproduce all results (within “AffineCa2p_reproduce_results” folder) were submitted as Extended Data. Each folder contains a readme file. Download Extended Data, EPS file. [file enu-eN-MNT-0054-20-s02.zip › data_code/AffineCa2p_reproduce_results/A5/reg_day17A5.png]

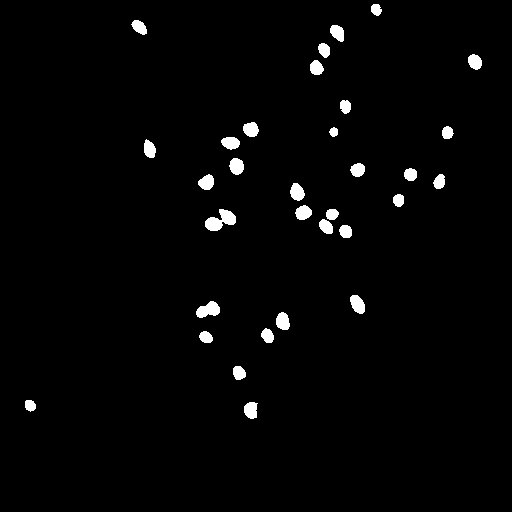

Supplement: Extended Data. — A zip file (named “data_code.zip”), including PyPI package (“FAIM_package” folder), example FOV images (within “examples” folder), and codes used to reproduce all results (within “AffineCa2p_reproduce_results” folder) were submitted as Extended Data. Each folder contains a readme file. Download Extended Data, EPS file. [file enu-eN-MNT-0054-20-s02.zip › data_code/AffineCa2p_reproduce_results/A6/raw_common_neurons/reg/ROI_day08_frame.tiff]

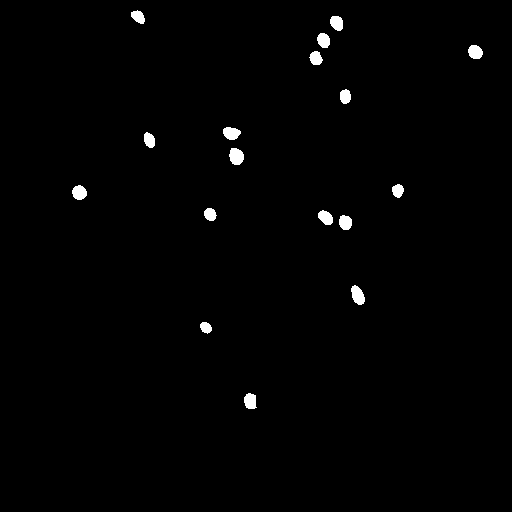

Supplement: Extended Data. — A zip file (named “data_code.zip”), including PyPI package (“FAIM_package” folder), example FOV images (within “examples” folder), and codes used to reproduce all results (within “AffineCa2p_reproduce_results” folder) were submitted as Extended Data. Each folder contains a readme file. Download Extended Data, EPS file. [file enu-eN-MNT-0054-20-s02.zip › data_code/AffineCa2p_reproduce_results/A6/raw_common_neurons/reg/ROI_day09_frame.tiff]

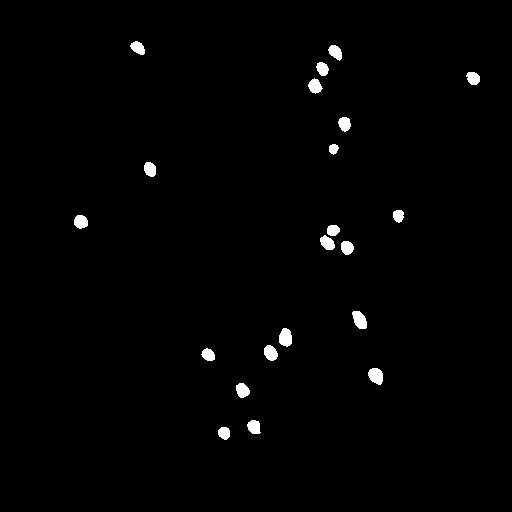

Supplement: Extended Data. — A zip file (named “data_code.zip”), including PyPI package (“FAIM_package” folder), example FOV images (within “examples” folder), and codes used to reproduce all results (within “AffineCa2p_reproduce_results” folder) were submitted as Extended Data. Each folder contains a readme file. Download Extended Data, EPS file. [file enu-eN-MNT-0054-20-s02.zip › data_code/AffineCa2p_reproduce_results/A6/raw_common_neurons/reg/ROI_day10_frame.tiff]

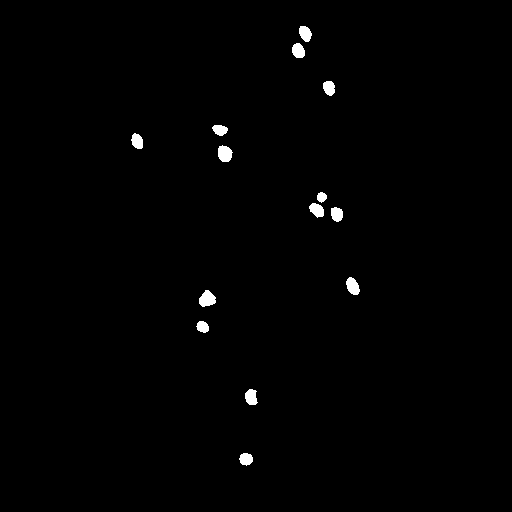

Supplement: Extended Data. — A zip file (named “data_code.zip”), including PyPI package (“FAIM_package” folder), example FOV images (within “examples” folder), and codes used to reproduce all results (within “AffineCa2p_reproduce_results” folder) were submitted as Extended Data. Each folder contains a readme file. Download Extended Data, EPS file. [file enu-eN-MNT-0054-20-s02.zip › data_code/AffineCa2p_reproduce_results/A6/raw_common_neurons/reg/ROI_day11_frame.tiff]

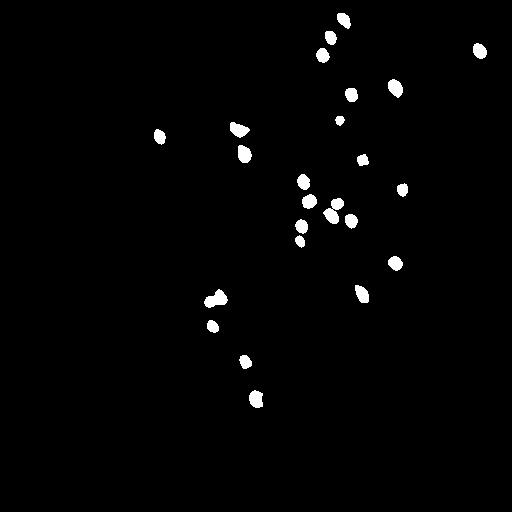

Supplement: Extended Data. — A zip file (named “data_code.zip”), including PyPI package (“FAIM_package” folder), example FOV images (within “examples” folder), and codes used to reproduce all results (within “AffineCa2p_reproduce_results” folder) were submitted as Extended Data. Each folder contains a readme file. Download Extended Data, EPS file. [file enu-eN-MNT-0054-20-s02.zip › data_code/AffineCa2p_reproduce_results/A6/raw_common_neurons/reg/ROI_day12_frame.tiff]

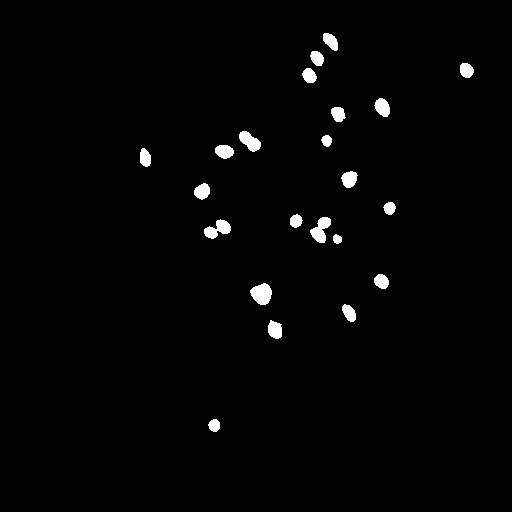

Supplement: Extended Data. — A zip file (named “data_code.zip”), including PyPI package (“FAIM_package” folder), example FOV images (within “examples” folder), and codes used to reproduce all results (within “AffineCa2p_reproduce_results” folder) were submitted as Extended Data. Each folder contains a readme file. Download Extended Data, EPS file. [file enu-eN-MNT-0054-20-s02.zip › data_code/AffineCa2p_reproduce_results/A6/raw_common_neurons/reg/ROI_day16_frame.tiff]

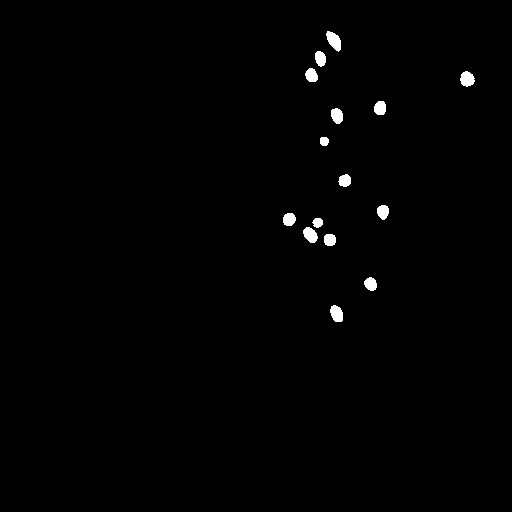

Supplement: Extended Data. — A zip file (named “data_code.zip”), including PyPI package (“FAIM_package” folder), example FOV images (within “examples” folder), and codes used to reproduce all results (within “AffineCa2p_reproduce_results” folder) were submitted as Extended Data. Each folder contains a readme file. Download Extended Data, EPS file. [file enu-eN-MNT-0054-20-s02.zip › data_code/AffineCa2p_reproduce_results/A6/raw_common_neurons/reg/ROI_day17_frame.tiff]

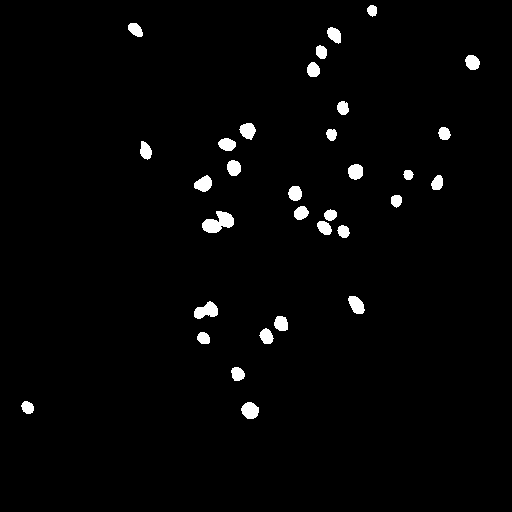

Supplement: Extended Data. — A zip file (named “data_code.zip”), including PyPI package (“FAIM_package” folder), example FOV images (within “examples” folder), and codes used to reproduce all results (within “AffineCa2p_reproduce_results” folder) were submitted as Extended Data. Each folder contains a readme file. Download Extended Data, EPS file. [file enu-eN-MNT-0054-20-s02.zip › data_code/AffineCa2p_reproduce_results/A6/raw_common_neurons/template/ROI_day06_08_frame.tiff]

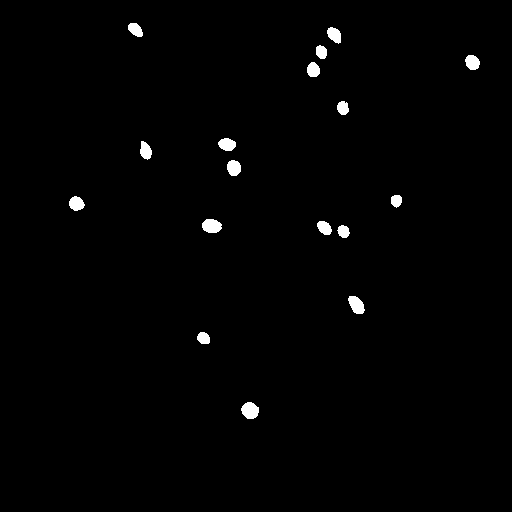

Supplement: Extended Data. — A zip file (named “data_code.zip”), including PyPI package (“FAIM_package” folder), example FOV images (within “examples” folder), and codes used to reproduce all results (within “AffineCa2p_reproduce_results” folder) were submitted as Extended Data. Each folder contains a readme file. Download Extended Data, EPS file. [file enu-eN-MNT-0054-20-s02.zip › data_code/AffineCa2p_reproduce_results/A6/raw_common_neurons/template/ROI_day06_09_frame.tiff]

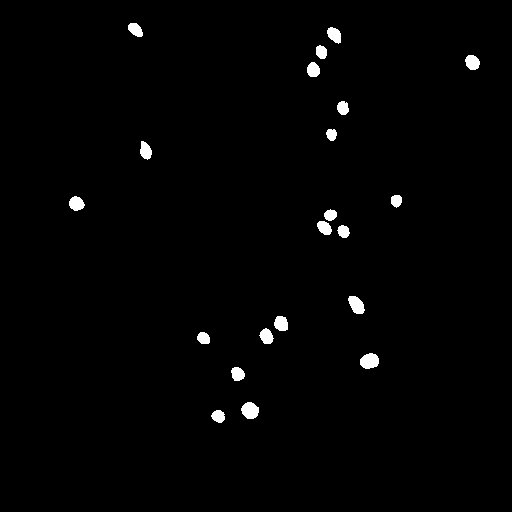

Supplement: Extended Data. — A zip file (named “data_code.zip”), including PyPI package (“FAIM_package” folder), example FOV images (within “examples” folder), and codes used to reproduce all results (within “AffineCa2p_reproduce_results” folder) were submitted as Extended Data. Each folder contains a readme file. Download Extended Data, EPS file. [file enu-eN-MNT-0054-20-s02.zip › data_code/AffineCa2p_reproduce_results/A6/raw_common_neurons/template/ROI_day06_10_frame.tiff]

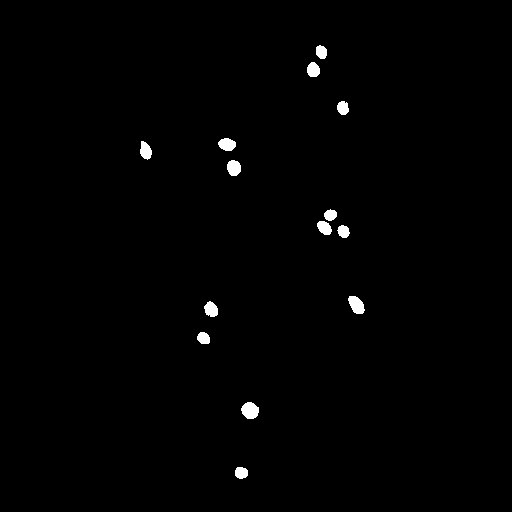

Supplement: Extended Data. — A zip file (named “data_code.zip”), including PyPI package (“FAIM_package” folder), example FOV images (within “examples” folder), and codes used to reproduce all results (within “AffineCa2p_reproduce_results” folder) were submitted as Extended Data. Each folder contains a readme file. Download Extended Data, EPS file. [file enu-eN-MNT-0054-20-s02.zip › data_code/AffineCa2p_reproduce_results/A6/raw_common_neurons/template/ROI_day06_11_frame.tiff]

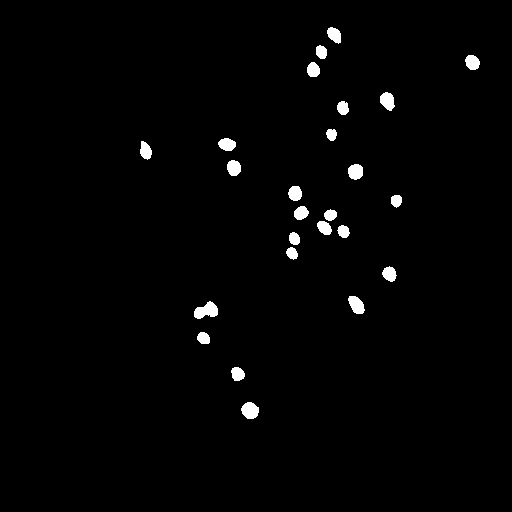

Supplement: Extended Data. — A zip file (named “data_code.zip”), including PyPI package (“FAIM_package” folder), example FOV images (within “examples” folder), and codes used to reproduce all results (within “AffineCa2p_reproduce_results” folder) were submitted as Extended Data. Each folder contains a readme file. Download Extended Data, EPS file. [file enu-eN-MNT-0054-20-s02.zip › data_code/AffineCa2p_reproduce_results/A6/raw_common_neurons/template/ROI_day06_12_frame.tiff]

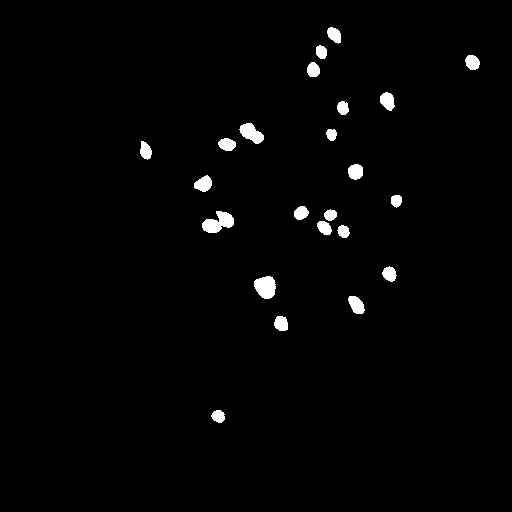

Supplement: Extended Data. — A zip file (named “data_code.zip”), including PyPI package (“FAIM_package” folder), example FOV images (within “examples” folder), and codes used to reproduce all results (within “AffineCa2p_reproduce_results” folder) were submitted as Extended Data. Each folder contains a readme file. Download Extended Data, EPS file. [file enu-eN-MNT-0054-20-s02.zip › data_code/AffineCa2p_reproduce_results/A6/raw_common_neurons/template/ROI_day06_16_frame.tiff]

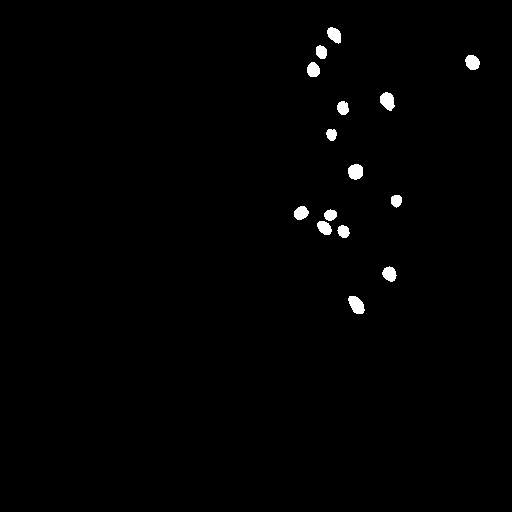

Supplement: Extended Data. — A zip file (named “data_code.zip”), including PyPI package (“FAIM_package” folder), example FOV images (within “examples” folder), and codes used to reproduce all results (within “AffineCa2p_reproduce_results” folder) were submitted as Extended Data. Each folder contains a readme file. Download Extended Data, EPS file. [file enu-eN-MNT-0054-20-s02.zip › data_code/AffineCa2p_reproduce_results/A6/raw_common_neurons/template/ROI_day06_17_frame.tiff]

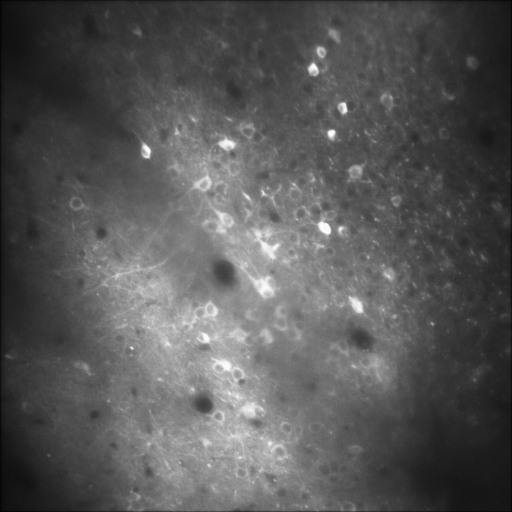

Supplement: Extended Data. — A zip file (named “data_code.zip”), including PyPI package (“FAIM_package” folder), example FOV images (within “examples” folder), and codes used to reproduce all results (within “AffineCa2p_reproduce_results” folder) were submitted as Extended Data. Each folder contains a readme file. Download Extended Data, EPS file. [file enu-eN-MNT-0054-20-s02.zip › data_code/AffineCa2p_reproduce_results/A6/reg_day06A6.png]

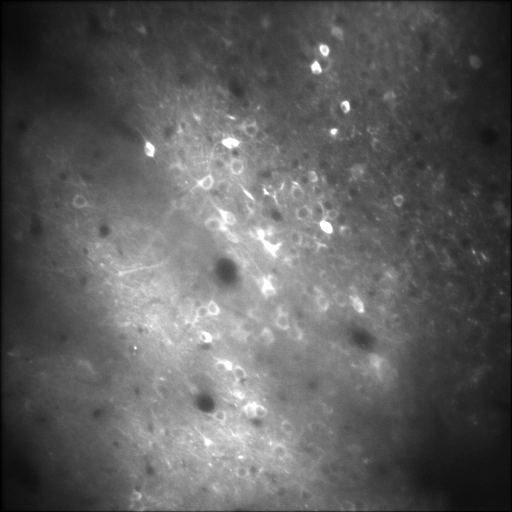

Supplement: Extended Data. — A zip file (named “data_code.zip”), including PyPI package (“FAIM_package” folder), example FOV images (within “examples” folder), and codes used to reproduce all results (within “AffineCa2p_reproduce_results” folder) were submitted as Extended Data. Each folder contains a readme file. Download Extended Data, EPS file. [file enu-eN-MNT-0054-20-s02.zip › data_code/AffineCa2p_reproduce_results/A6/reg_day08A6.png]

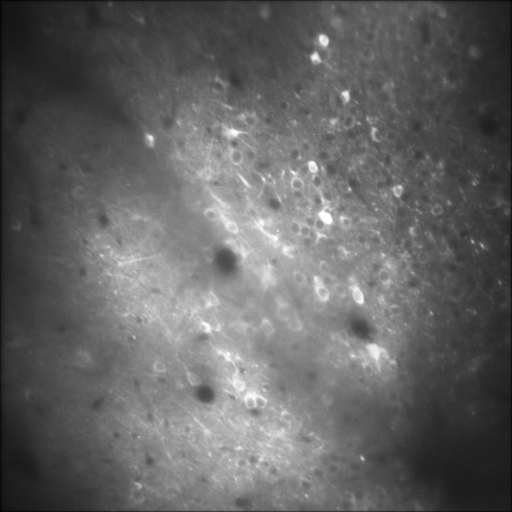

Supplement: Extended Data. — A zip file (named “data_code.zip”), including PyPI package (“FAIM_package” folder), example FOV images (within “examples” folder), and codes used to reproduce all results (within “AffineCa2p_reproduce_results” folder) were submitted as Extended Data. Each folder contains a readme file. Download Extended Data, EPS file. [file enu-eN-MNT-0054-20-s02.zip › data_code/AffineCa2p_reproduce_results/A6/reg_day09A6.png]

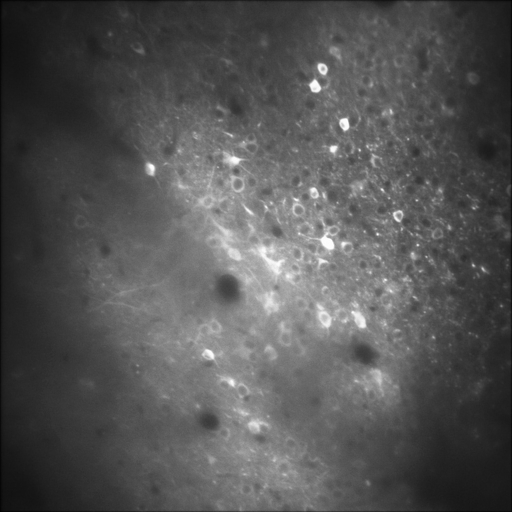

Supplement: Extended Data. — A zip file (named “data_code.zip”), including PyPI package (“FAIM_package” folder), example FOV images (within “examples” folder), and codes used to reproduce all results (within “AffineCa2p_reproduce_results” folder) were submitted as Extended Data. Each folder contains a readme file. Download Extended Data, EPS file. [file enu-eN-MNT-0054-20-s02.zip › data_code/AffineCa2p_reproduce_results/A6/reg_day10A6.png]

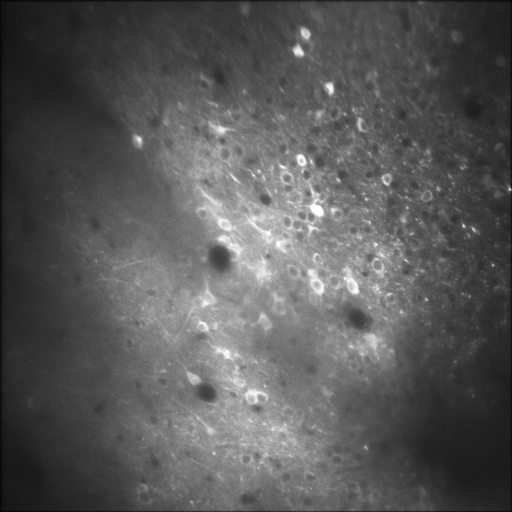

Supplement: Extended Data. — A zip file (named “data_code.zip”), including PyPI package (“FAIM_package” folder), example FOV images (within “examples” folder), and codes used to reproduce all results (within “AffineCa2p_reproduce_results” folder) were submitted as Extended Data. Each folder contains a readme file. Download Extended Data, EPS file. [file enu-eN-MNT-0054-20-s02.zip › data_code/AffineCa2p_reproduce_results/A6/reg_day11A6.png]

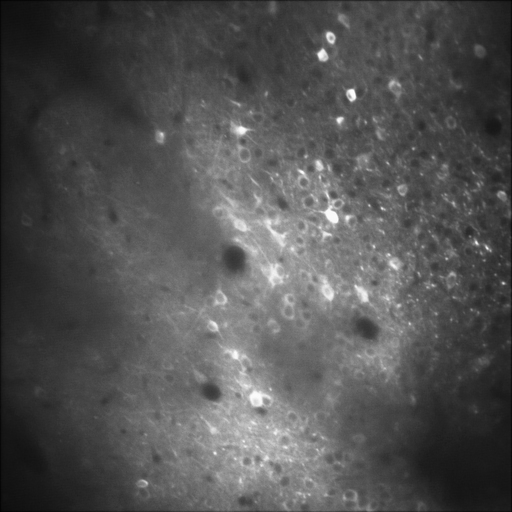

Supplement: Extended Data. — A zip file (named “data_code.zip”), including PyPI package (“FAIM_package” folder), example FOV images (within “examples” folder), and codes used to reproduce all results (within “AffineCa2p_reproduce_results” folder) were submitted as Extended Data. Each folder contains a readme file. Download Extended Data, EPS file. [file enu-eN-MNT-0054-20-s02.zip › data_code/AffineCa2p_reproduce_results/A6/reg_day12A6.png]

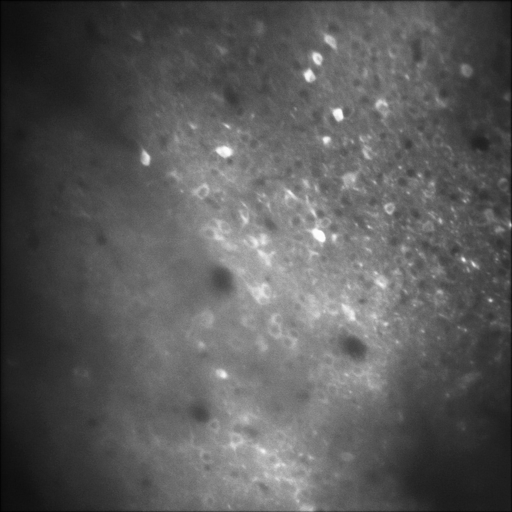

Supplement: Extended Data. — A zip file (named “data_code.zip”), including PyPI package (“FAIM_package” folder), example FOV images (within “examples” folder), and codes used to reproduce all results (within “AffineCa2p_reproduce_results” folder) were submitted as Extended Data. Each folder contains a readme file. Download Extended Data, EPS file. [file enu-eN-MNT-0054-20-s02.zip › data_code/AffineCa2p_reproduce_results/A6/reg_day16A6.png]

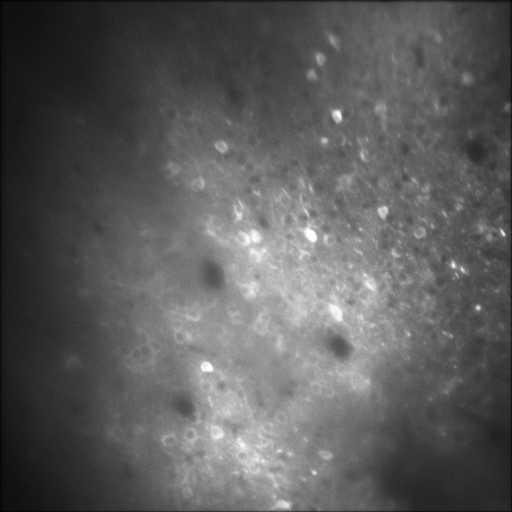

Supplement: Extended Data. — A zip file (named “data_code.zip”), including PyPI package (“FAIM_package” folder), example FOV images (within “examples” folder), and codes used to reproduce all results (within “AffineCa2p_reproduce_results” folder) were submitted as Extended Data. Each folder contains a readme file. Download Extended Data, EPS file. [file enu-eN-MNT-0054-20-s02.zip › data_code/AffineCa2p_reproduce_results/A6/reg_day17A6.png]

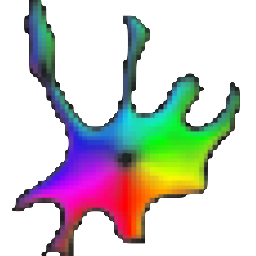

Supplement: Extended Data. — A zip file (named “data_code.zip”), including PyPI package (“FAIM_package” folder), example FOV images (within “examples” folder), and codes used to reproduce all results (within “AffineCa2p_reproduce_results” folder) were submitted as Extended Data. Each folder contains a readme file. Download Extended Data, EPS file. [file enu-eN-MNT-0054-20-s02.zip › data_code/AffineCa2p_reproduce_results/cellpose/logo/logo.png]

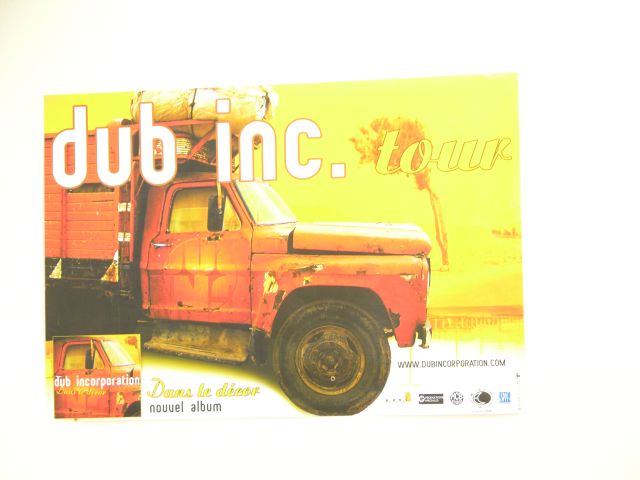

Supplement: Extended Data. — A zip file (named “data_code.zip”), including PyPI package (“FAIM_package” folder), example FOV images (within “examples” folder), and codes used to reproduce all results (within “AffineCa2p_reproduce_results” folder) were submitted as Extended Data. Each folder contains a readme file. Download Extended Data, EPS file. [file enu-eN-MNT-0054-20-s02.zip › data_code/AffineCa2p_reproduce_results/matlab_codes/IAT_v0.9.3/dirt/source.jpg]

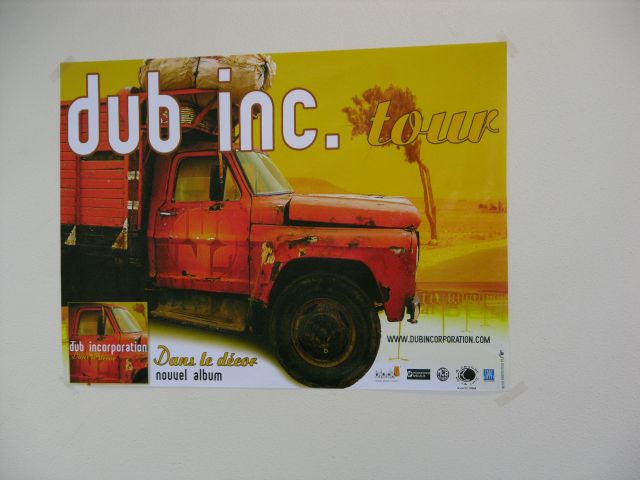

Supplement: Extended Data. — A zip file (named “data_code.zip”), including PyPI package (“FAIM_package” folder), example FOV images (within “examples” folder), and codes used to reproduce all results (within “AffineCa2p_reproduce_results” folder) were submitted as Extended Data. Each folder contains a readme file. Download Extended Data, EPS file. [file enu-eN-MNT-0054-20-s02.zip › data_code/AffineCa2p_reproduce_results/matlab_codes/IAT_v0.9.3/dirt/target.jpg]
